# Multidomain Outcomes of a Digital Self-Management Program in Rural Adults With Chronic Conditions: Single-Arm Prospective Interventional Study

**DOI:** 10.2196/95283

**Published:** 2026-10-30

**Authors:** Nathan K Smith, Gabriela Ilie, Cody MacDonald, James Richard Ballentyne Langley, Robert David Harold Rutledge

**Affiliations:** 1 Department of Community Health and Epidemiology, Faculty of Medicine, Dalhousie University, 5790 University Avenue, Halifax, NS, B3H 1V7, Canada, 1 9029894114; 2 Department of Urology, Faculty of Medicine, Dalhousie University, Halifax, NS, Canada; 3 Department of Radiation Oncology, Faculty of Medicine, Dalhousie University, Halifax, NS, Canada; 4 Health Sciences Department, Queen's University, Kingston, ON, Canada

**Keywords:** rural health services, mental health, patient empowerment, home-based interventions, chronic disease, psychological distress

## Abstract

**Background:**

Chronic disease and comorbid mental health problems disproportionately affect rural and underserved populations. Digital self-management programs are potentially scalable, but evaluations often focus on narrow outcomes.

**Objective:**

This study examined 12-month longitudinal changes in psychological and multidomain outcomes among Pictou County Patient Empowerment Program participants and explored co-occurring changes and psychological moderators.

**Methods:**

This single-arm prospective interventional study enrolled 182 community-dwelling adults with ≥1 chronic condition in Pictou County, Nova Scotia, between October 2023 and August 2025 through community-based outreach. Median age was 60 (IQR 51‐68) years, 155 of 182 (85.2%) participants were women, and the median number of chronic conditions was 3 (IQR 1‐3). The initial 6-month digital intervention delivered brief daily modules targeting physical activity, nutrition, stress regulation, sleep, habit formation, and social connection, followed by a 6-month lower-intensity maintenance period during which monthly group videoconference sessions continued to be offered. Outcomes were assessed at baseline, 6 months, and 12 months. Primary outcomes were illness perceptions (Brief Illness Perception Questionnaire), perceived stress (10-item Perceived Stress Scale [PSS-10]), disease-related worry (10-item Questionnaire on Distress in Cancer Patients), life satisfaction (Satisfaction With Life Scale), and mental well-being (World Health Organization-Five Well-Being Index [WHO-5]). Secondary outcomes assessed the physical, behavioral, and psychosocial domains. Generalized estimating equations estimated population-averaged longitudinal changes, adjusted for age, sex, relationship status, comorbidity burden, and baseline depression or anxiety medication use. Exploratory models examined co-occurring changes and moderation. Tests were 2-sided with *α*=.05.

**Results:**

Across outcomes and follow-up assessments, completion ranged from 58.2% (95% CI 51.0%-65.2%; 106/182) to 65.4% (95% CI 58.2%‐71.9%; 119/182). At 12 months, illness perceptions decreased (β=−7.3, 95% CI −9.7 to −4.9), perceived stress decreased (β=−2.4, 95% CI −3.5 to −1.3), disease-related worry decreased (β=−6.3, 95% CI −7.8 to −4.7), life satisfaction increased (β=2.5, 95% CI 1.4‐3.5), and mental well-being increased (β=10.4, 95% CI 7.2‐13.5; all *P*<.001). Secondary outcomes improved across fatigue, sleep, diet quality, sedentary behavior, spiritual well-being, mental health–related quality of life, weight, and BMI. Exploratory analyses showed that improvements in fatigue, sleep quality, and illness perceptions co-occurred with greater well-being and life satisfaction and lower perceived stress and disease-related worry at both follow-up assessment points. Clinically meaningful stress improvement (≥4-point PSS-10 reduction) was achieved by 36.4% (95% CI 27.3%-45.6%, 39/107) and 43.4% (95% CI 34.0%-52.8%, 46/106) participants at 6 and 12 months, respectively. At 12 months, 43.5% (95% CI 34.2%-52.9%, 47/108) participants reported clinically meaningful improvement in mental well-being (≥10-point WHO-5 increase).

**Conclusions:**

Participation in this low-bandwidth, home-based digital self-management program was associated with sustained psychological, physical, and behavioral improvements among rural adults with chronic conditions. These multidomain findings support randomized controlled evaluation of scalable digital self-management approaches as adjuncts to rural chronic disease care.

## Introduction

Chronic disease represents a growing public health challenge. In 2021, approximately 45% of Canadians were living with at least 1 major chronic condition, and 1 in 12 had 3 or more comorbid chronic diseases [1]. Globally, chronic diseases account for an estimated 75% of non–pandemic-related deaths and are projected to generate cumulative health care costs exceeding US $47 trillion by 2030 [2,3]. Chronic disease frequently co-occurs with adverse mental health outcomes, including depression, anxiety, and psychological distress, contributing to reduced quality of life, symptom exacerbation, and higher risk of downstream morbidity and mortality [4,5].

Rural populations may be particularly vulnerable to this clustering of physical and mental health burden. Compared with urban populations, rural residents experience a higher prevalence of chronic disease [6] and face substantial barriers to accessing specialized care, including mental health services. Even among individuals prescribed pharmacological treatment for mental health conditions, access to follow-up and supportive services declines across urban, rural-adjacent, and rural-nonadjacent settings [7]. These disparities underscore the need for accessible, scalable approaches to chronic disease management that extend beyond disease-specific biomedical care.

Effective self-management is a central component of chronic disease care. Clinical practice guidelines increasingly emphasize self-management education and support as core elements of long-term disease control [8]. Evidence from systematic reviews and meta-analyses demonstrates that self-management programs improve quality of life, self-efficacy, and depressive symptoms [9]; that interventions incorporating psychotherapeutic components yield superior physiological outcomes [10]; and that multicomponent approaches targeting multiple behaviors simultaneously are more effective than single-behavior interventions [11]. Despite this evidence, many chronic disease programs omit outcomes and supports that patients identify as important, including stress regulation, emotional well-being, and social connection [12]. Social support in particular influences self-management both directly and indirectly through empowerment and psychological resilience [13]. Even in the context of emerging pharmacotherapies for obesity-related and cardiometabolic conditions, experts increasingly emphasize the need for integrated psychosocial and behavioral support to sustain benefits and mitigate adverse psychological effects [14].

Although many chronic diseases are preventable or modifiable, health systems remain largely organized around reactive, episodic care, prioritizing treatment of acute disease escalation rather than sustained self-management and prevention. Models such as the Expanded Chronic Care Model emphasize proactive support, patient activation, and community-based resources, yet scalable implementations of these principles remain limited, particularly for populations living with multimorbidity and in rural settings. As a result, many individuals experience fragmented care that addresses downstream symptoms without adequately targeting upstream behavioral and psychosocial processes that shape chronic disease trajectories over time.

Digital health interventions offer a promising and scalable approach to address these gaps [15,16]. Importantly, digital self-management programs can extend behavioral and psychosocial support beyond traditional clinical encounters by embedding brief, scalable guidance into patients’ daily routines, enabling continuous engagement with health behaviors between health care visits. Such interventions can extend care beyond traditional clinical encounters; support continuity and personalization; and incorporate outcomes that matter to patients, including sleep, physical activity, and psychosocial well-being [15,17]. By addressing shared behavioral and psychosocial risk factors across conditions, digital self-management programs have the potential to activate patients in their own care, support daily decision-making, and improve adherence and longer-term outcomes.

While digital interventions for chronic disease have demonstrated benefits for mental health outcomes, the mechanisms through which these improvements occur remain poorly understood. Meta-analyses of digital interventions for depression suggest that change occurs through multiple indirect pathways, including cognitive, behavioral, self-related, physiological, and mindfulness-related processes [18,19]. However, most individual studies are narrow in scope, mechanistic insights outside depression are limited, and few evaluations examine multidomain change over extended periods. Mechanistic understanding is particularly sparse in rural populations and in digital interventions addressing chronic disease more broadly. Identifying co-occurring changes across psychological, physical, behavioral, and social domains, as well as factors that modify response, is essential for optimizing intervention design and implementation.

To address these gaps, the Pictou County Patient Empowerment Program (PictouCountyPEP) was developed as a 6-month, comprehensive digital self-management program for adults living with chronic conditions in a rural setting [20]. A prior digital health evaluation established the feasibility, usability, and sustained mental health benefits of this low-bandwidth delivery model in rural adults with chronic disease [20]. The present study extends this work by evaluating longer-term change across psychological, physical, behavioral, and social domains and by examining whether improvements occur in theoretically coherent patterns relevant to chronic disease self-management.

The program builds on an empowerment-based intervention model previously evaluated in cancer populations [21,22] and was adapted to remove cancer-specific content while retaining the core behavioral and psychosocial components. Rather than targeting a single symptom or disease-specific pathway, the program was designed to influence modifiable upstream processes related to stress regulation, coping, self-efficacy, health behaviors, and social connection that are expected to covary with psychological distress and chronic disease burden over time. Chronic diseases frequently cluster and progress through shared upstream processes, including stress dysregulation, physical inactivity, sleep disruption, social isolation, and reduced self-efficacy. Interventions that target these shared processes may be preventive in orientation, with potential to attenuate escalation and accumulation of multimorbidity, even when not designed to prevent specific diagnoses.

The primary objective of this study was to quantify longitudinal changes in psychological outcomes over 12 months following participation in PictouCountyPEP. Concurrent changes in physical, behavioral, and social outcomes were examined to contextualize psychological change. Secondary objectives were to characterize changes across physical, behavioral, and social outcomes; examine whether changes in selected candidate factors were associated with changes in psychological outcomes; and explore whether baseline psychological characteristics modified longitudinal change. These analyses were exploratory and hypothesis-generating and were intended to identify candidate pathways for evaluation in future controlled studies rather than establish causal mechanisms.

Understanding longitudinal psychosocial, physical, and behavioral changes associated with participation in a comprehensive digital self-management program can inform clinical decision-making in rural primary care, support health system planning for chronic disease management, and guide adaptation for populations at higher risk of multimorbidity. Identifying potential psychological pathways of change may further inform the optimization, personalization, and scale-up of digital empowerment interventions in rural and underserved regions.

## Methods

### Study Design

This study reports outcomes from the PictouCountyPEP, a single-arm, prospective, 6-month digital behavioral intervention delivered to adults living with chronic health conditions in Pictou County, Nova Scotia, Canada (October 2023 – August 2025). The study was designed as a pragmatic, real-world evaluation of feasibility, engagement, and longitudinal change under community delivery conditions rather than as a controlled efficacy trial; accordingly, observed changes over time cannot be attributed solely to the intervention.

### Setting

Pictou County is a predominantly rural region with approximately 44,000 residents and limited access to specialty health services [23].

### Participant Inclusion and Exclusion Criteria

Adults aged 18 years or older residing in Pictou County and living with 1 or more self-reported chronic conditions were eligible to participate. Eligibility was not restricted to a prespecified list of chronic disease diagnoses; the program was intentionally designed for adults living with chronic conditions across diagnostic categories and for those with multimorbidity. Eligibility required the ability to safely engage in low- to moderate-intensity physical activity, assessed using the Physical Activity Readiness Questionnaire for Everyone (PAR-Q+) [24], English proficiency, and access to the internet and email. No specific chronic disease categories were otherwise excluded; however, individuals with a current or recent cancer diagnosis were excluded because the intervention model has been evaluated separately in cancer populations in prior trials [21,22].

### Recruitment and Sampling Procedures

Recruitment occurred through community-based outreach, including local media, social media, flyers, and partner organizations [20]. Eligible participants were enrolled consecutively as they presented for participation during the recruitment period.

### Ethical Considerations

Participants provided electronic informed consent via the REDCap eConsent framework. No financial compensation for study participation was provided. Data were collected and managed in accordance with Nova Scotia Health privacy policies and the requirements of Canada’s Personal Information Protection and Electronic Documents Act (PIPEDA). No images identifying individual participants are included in the manuscript or supplementary materials. The study was approved by the Nova Scotia Health Research Ethics Board (REB 1029744) prior to participant enrollment and was registered on ClinicalTrials.gov (NCT06064110).

### Data Collection and Assessment Schedule

Self-reported surveys were administered using REDCap [25]. Participants completed assessments at baseline, 6 months, and 12 months.

### Intervention

PictouCountyPEP is a home-based, multicomponent digital intervention designed to support self-management and mental well-being among adults living with chronic health conditions. The intervention was adapted from previously evaluated patient empowerment program models and modified to remove all cancer-specific education. The full protocol has been published previously [20].

The program delivered daily automated emails over a 26-week period, linking participants to brief instructional videos hosted by authors GI and RDHR. The videos were approximately 4 to 6 minutes in duration and accessible asynchronously via a public video-hosting platform. The content addressed 6 behavioral domains: aerobic physical activity, strength training or yoga, relaxation and stress management, dietary guidance, habit formation, and relationship and social connection skills. The content was modular, allowing flexible engagement according to individual needs and symptom burden.

Exercise guidance aligned with the national physical activity recommendations, encouraging 150 to 300 minutes per week of moderate-intensity aerobic activity and strength or flexibility training at least twice weekly. The content emphasized pacing, gradual progression, and symptom monitoring, with encouragement to tailor activities in consultation with health care providers. Dietary guidance promoted a predominantly plant-based pattern with reduced intake of ultra-processed foods, consistent with Canada’s Food Guide [26]. The stress management content incorporated mindfulness-based practices, breathing techniques, and cognitive strategies. The relationship modules focused on communication, boundary setting, and social connection, while the habit formation content emphasized small, sustainable behavioral changes.

The intervention was designed for low-bandwidth delivery and mobile accessibility to minimize barriers related to internet speed, device availability, or digital literacy. No specialized applications, wearable devices, or synchronous participation was required. Engagement was encouraged through daily reminders and weekly compliance surveys. Optional components included a peer support option, monthly live group videoconference sessions, and occasional in-person community events. These components were offered to enhance social connection and accountability but were not required or standardized. The intervention was delivered under real-world conditions to maximize external validity and inform future scale-up. Given the single-arm interventional design, this study follows the TREND (Transparent Reporting of Evaluations with Nonrandomized Designs) guidelines for nonrandomized evaluations of behavioral interventions [27]. The daily digital intervention content was delivered during the initial 6 months. During months 7 to 12, participants entered a lower-intensity maintenance period in which monthly group videoconference sessions continued to be offered, but daily intervention emails were no longer delivered. The 12-month assessment therefore reflects outcomes following the initial 6-month daily intervention and the subsequent 6-month lower-intensity maintenance period.

Because the intervention content was delivered asynchronously and participants could engage flexibly with individual components, standardized individual-level measures of total intervention exposure or module completion were not available for the present analysis; consequently, a formal dose-response analysis was not conducted.

### Outcomes and Measures

#### Primary and Secondary Outcomes and Measures

Primary outcomes were prespecified psychological measures reflecting illness perceptions, well-being, perceived stress, life satisfaction, and disease-related worry. Participants completed validated self-report measures assessing psychological, physical, behavioral, and social outcomes. All multi-item composite scales were scored using complete item-level data. References for all measures and cut points can be found in the Multimedia Appendix 1.

#### Primary Psychological Measures

Illness perceptions were assessed using the 9-item Brief Illness Perception Questionnaire (B-IPQ), which measures the perceived consequences, timeline, personal control, treatment control, symptom identity, concern, emotional response, and illness coherence related to chronic illness [28]. Each item is rated on a 0 to 10 numeric scale, with 3 items being reverse-coded. Higher scores indicate more threatening or negative illness perceptions. A total score was derived by summing all items, and an improvement of ≥7 was considered clinically meaningful [29].

Psychological well-being was measured using the World Health Organization-Five Well-Being Index (WHO-5), a 5-item instrument assessing positive mood, vitality, and general interest in daily life over the preceding 2 weeks [30]. Raw scores (range 0‐25) were multiplied by 4 to yield a total score ranging from 0 to 100, with higher scores indicating better well-being. Scores below 50 were used to indicate poor well-being in binary analyses [31].

Perceived stress was assessed using the 10-item Perceived Stress Scale (PSS-10), which measures the extent to which situations in one’s life are appraised as stressful, unpredictable, and uncontrollable during the past month [32]. Items are rated from 0 (“never”) to 4 (“very often”), with 4 items being reverse-coded. The items were summed to produce a total score ranging from 0 to 40, with higher scores indicating greater perceived stress. Scores ≥20 were used to classify high perceived stress, consistent with prior studies [33].

Life satisfaction was measured using the Satisfaction With Life Scale (SWLS), a 5-item instrument assessing global cognitive judgments of satisfaction with one’s life [34]. Items are rated from 1 (“strongly disagree”) to 7 (“strongly agree”) and summed to yield total scores ranging from 5 to 35, with higher scores indicating greater life satisfaction. For responder analyses, an improvement of ≥5 points was used as an interpretive threshold informed by the scale author’s score categories [35].

Disease-related worry was assessed using the 10-item Questionnaire on Distress in Cancer Patients (QSC-R10), which measures illness-related fears and concerns relevant to individuals living with chronic conditions [36]. Each item assesses whether a concern applies and the degree of distress it causes, rated from 0 to 5. Items were summed to produce total scores ranging from 0 to 50, with higher scores indicating greater disease-related worry. A cut point of>14 was used to classify clinically relevant need for psychosocial support in exploratory analyses [36].

#### Secondary Physical, Psychosocial, and Behavioral Measures

BMI was derived from self-reported weight and height. BMI (kg/m^2^) was calculated as weight (kg) divided by height (m) squared. Outcomes included continuous change in weight (lb) and BMI, as well as categorical indicators of clinically meaningful weight loss (≥3%), consistent with previous trials and recommended guidelines [37,38].

Health-related quality of life was assessed using the 12-item Short Form Health Survey (SF-12), yielding norm-based Physical Component Summary (PCS) and Mental Component Summary (MCS) scores standardized to a mean of 50 and SD of 10 in the general population [39,40]. Higher scores indicate better health-related quality of life. Score changes of ≥3 points were considered clinically meaningful, in line with previous research [41].

Fatigue was assessed using the 13-item Functional Assessment of Chronic Illness Therapy—Fatigue scale (FACIT-F) [42], which evaluates fatigue severity and its impact on daily functioning. Items are rated from 0 (“not at all”) to 4 (“very much”) and summed after reverse scoring, such that higher scores indicate less fatigue. A change in score of ≥3 points was used to define clinically meaningful improvement [43].

Sleep quality was measured using the Pittsburgh Sleep Quality Index (PSQI), which yields a global score ranging from 0 to 21, with higher scores indicating poorer sleep quality [44]. A global score >5 was used to identify poor sleep [44].

Sedentary behavior was assessed using the adult Sedentary Behavior Questionnaire (SBQ), which captures time spent in common sedentary activities during weekdays and weekends [45]. Reported hours were converted to total daily sedentary time, with values exceeding 24 hours being truncated. Daily weekday and weekend hours were summed to obtain weekly sedentary hours. High sedentary behavior was defined as ≥8 hours per day (equivalent to ≥56 hours per week), consistent with prior studies [46].

Perceived social support was measured using the 19-item Medical Outcomes Study Social Support Survey (MOS-SSS), yielding emotional and informational, tangible, affectionate, positive interaction, and overall support scores [47]. Items are rated from 1 to 5 and averaged according to scoring guidelines, with higher scores indicating greater perceived support.

Diet quality was assessed using the Rapid Eating Assessment for Participants—Shortened version (REAP-S), a 13-item measure of dietary habits over the past week [48]. Items are scored from 1 to 3, yielding total scores ranging from 13 to 39, with higher scores indicating healthier dietary patterns.

Spiritual well-being was measured using the 12-item Functional Assessment of Chronic Illness Therapy—Spiritual Well-Being scale (FACIT-SP-12), which assesses meaning, peace, and faith [49]. Items are scored from 0 to 4 (reversed where scoring instructions indicate) and summed to yield total scores ranging from 0 to 48, with higher scores indicating greater spiritual well-being.

Alcohol consumption was assessed using self-reported drinking and binge drinking frequency over the preceding 6 months. Binge drinking was defined as consuming 5 or more drinks containing any kind of alcohol within a 2-hour period [50]. Original categorical responses were converted into continuous frequency scores to retain variability and maximize statistical power, with values ranging from 0 (none) to 9 (daily) [51].

Cannabis use was assessed using self-reported frequency of use over the preceding 6 months and self-identification as a regular user. Categorical frequency responses were converted into a continuous score ranging from 0 (no use) to 4 (8 or more uses), using the same rationale applied to alcohol frequency scoring [51].

### Measurement Reliability

Internal consistency of all multi-item scales was assessed at baseline using Cronbach α with 95% CI to characterize measurement reliability in the study population prior to intervention exposure (Table S1 in Multimedia Appendix 1).

### Covariates

Covariates included age, sex, relationship status, comorbidity burden measured by the Charlson Comorbidity Index, and use of medication for depression or anxiety at baseline. These variables were selected a priori based on theoretical relevance and prior evidence from related patient empowerment program trials identifying their associations with mental health outcomes and intervention response [20-22].

### Sample Size, Power, and Precision

This phase 2 interventional study enrolled 182 participants to evaluate feasibility, engagement, and longitudinal changes across a broad set of health outcomes. The sample size was determined pragmatically based on anticipated recruitment capacity within the study region and time frame rather than a formal a priori power calculation, consistent with the study’s phase 2 implementation-focused objectives.

### Statistical Analysis

All participants who consented and completed baseline assessments were included in the analytic cohort. Primary longitudinal analyses incorporated all available observed outcome data from participants who contributed data at the relevant assessment time points.

Longitudinal changes in psychosocial, physical, and behavioral outcomes were examined using generalized estimating equations (GEEs), which estimate population-averaged effects while accounting for within-participant correlation across repeated measurements. Continuous outcomes were modeled using an identity link with Gaussian working variance, and binary outcomes defined using established clinical thresholds (Multimedia Appendix 1) were modeled using a logit link with binomial working variance. An exchangeable working correlation structure was specified for all models, reflecting the limited number of equally spaced follow-up assessments, and robust (sandwich) SEs were used for inference to ensure valid estimation under potential misspecification of the working correlation. GEEs were selected to estimate population-averaged effects aligned with the study objective of evaluating overall program-associated change rather than subject-specific trajectories.

Time was modeled as a categorical variable (baseline, 6 months, and 12 months). All models were adjusted a priori for baseline age, sex, relationship status, comorbidity burden (Charlson Comorbidity Index), and use of medication for depression or anxiety. Model-based estimated marginal means and corresponding SEs were derived at each time point, and pairwise contrasts were used to estimate differences between baseline and follow-up assessments, with 95% CI reported. Primary inference for continuous outcomes was based on time indicator coefficients from these models, representing adjusted population-averaged mean differences from baseline.

Exploratory interaction models were estimated for psychological outcomes only, by including baseline characteristics interacted with time, to examine whether baseline factors modified trajectories of psychological change. These analyses were prespecified as exploratory and interpreted as hypothesis-generating.

Exploratory analyses examined associations between within-participant changes in selected candidate factors and changes in psychological outcomes. These analyses were intended to identify patterns of co-occurring change and generate hypotheses regarding potential pathways rather than to test formal statistical mediation or establish causal mechanisms. Residualized change scores from baseline to follow-up were calculated, and linear regression models were fitted to assess associations between changes in physical, psychosocial, and substance use measures and changes in psychological well-being (WHO-5), perceived stress (PSS-10), life satisfaction (SWLS), and disease-related worry (QSC-R10). Models were adjusted for baseline outcome values and prespecified covariates. Results are interpreted as exploratory associations between longitudinal changes rather than as estimates of mediation or causal effects.

Clinically meaningful improvement was further examined using responder analyses based on established thresholds. Two responder definitions were applied: magnitude-based change, defined as achieving a prespecified minimally important difference regardless of baseline status, and categorical improvement, defined as movement from a clinically worse category at baseline to a more favorable category at follow-up, restricted to participants eligible to improve. Responder proportions at 6 and 12 months were calculated with 95% CI. Exploratory univariate logistic regression models were used to examine associations between baseline characteristics and responder status.

### Missing Data and Sensitivity Analyses

Missing follow-up data were evaluated using several complementary approaches. In unadjusted comparisons, participants with incomplete follow-up had significantly lower baseline well-being (WHO-5; *P*=.03) and life satisfaction (SWLS; *P*=.01) than participants with complete follow-up, whereas no statistically significant differences were observed for age, sex, comorbidity burden, relationship status, baseline depression/anxiety medication use, illness perceptions (B-IPQ), perceived stress (PSS-10), or disease-related worry (QSC-R10) (Table S2 in Multimedia Appendix 1). Complete follow-up was defined as completion of assessments at baseline, 6 months, and 12 months; follow-up was nonmonotonic, with some participants missing the 6-month assessment but returning at 12 months. Missingness was further evaluated using logistic regression models for incomplete follow-up, including all prespecified covariates and primary psychological outcomes as predictors. None of the included variables was statistically significantly associated with incomplete follow-up in the multivariable model. Primary analyses used covariate-adjusted GEE models and included all available observed outcome data, regardless of follow-up status. To evaluate the robustness of findings to incomplete follow-up, sensitivity analyses were conducted using both complete case analyses and analyses using stabilized inverse probability weights for attrition. Findings were highly consistent across the primary, complete case, and weighted analyses, indicating that the principal results were not materially altered by the analytic approach to incomplete follow-up.

All analyses were conducted using R software (version 4.5.1; R Foundation for Statistical Computing) [52]. Statistical tests were 2-sided, with a significance level of *α*=.05. Given the number of outcomes assessed, no formal adjustment for multiple comparisons was applied; results for secondary and exploratory analyses are therefore interpreted with emphasis on effect sizes, CI, and consistency of findings rather than statistical significance alone, and these findings are considered hypothesis-generating rather than confirmatory. Full model–based estimated marginal means and associated SEs for continuous outcomes are provided in Multimedia Appendix 1.

## Results

### Participants and Baseline Characteristics

A total of 182 individuals enrolled and completed the baseline assessment. Across outcomes and follow-up assessments, outcome-specific completion ranged from 106 of 182 (58.2%) participants to 119 of 182 (65.4%) participants (Figure 1; Table S4 in Multimedia Appendix 1). Baseline characteristics are shown in Table 1. Participants had a median age of 60 (IQR 51‐68) years; of the 182 participants, 155 (85.2%) were women, and 89 (48.9%) resided in the Municipality of Pictou County. The median number of diagnosed chronic conditions was 3 (IQR 1‐3), and the median age-adjusted Charlson Comorbidity Index was 2 (IQR 1‐3). The baseline symptom burden was substantial: of 182 participants, 52.7% (n=96) screened positive for low well-being (WHO-5<50), 41.2% (n=75) screened positive for high perceived stress (PSS-10≥20), and 48.4% (n=88) screened positive for psychological distress. Additional baseline sociodemographic, clinical, and health care use characteristics are reported in Table S3 in Multimedia Appendix 1. The follow-up status for each outcome is reported in Table S4 Multimedia Appendix 1.

**Figure 1.**
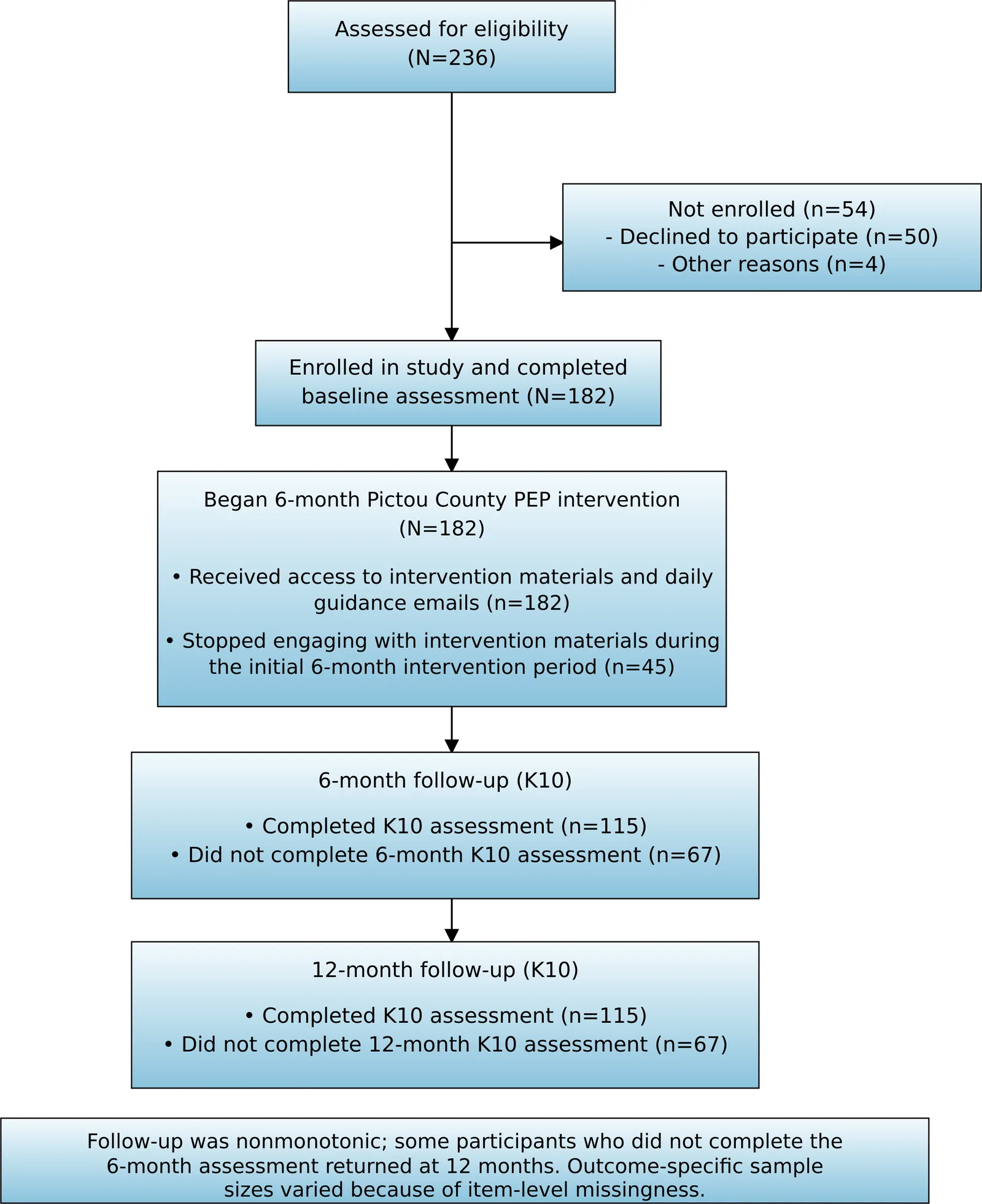
Participant flow is presented in the JARS-adapted participant flow diagram. JARS: Journal Article Reporting Standards; PEP: patient empowerment program.

**Table 1.** Baseline sociodemographic and clinical characteristics of participants enrolled in the Pictou County Patient Empowerment Program (N=182)^a^.

Characteristic	Value
Age (years), median (IQR)	60 (51-68)
Sex assigned at birth, n (%)	
Male	27 (14.8)
Female	155 (85.2)
Gender (self-report), n (%)	
Male	27 (14.8)
Female	155 (85.2)
Sexual orientation, n (%)	
Heterosexual	168 (92.3)
Gay (self-identified)	1 (0.6)
Bisexual	7 (3.8)
Other/unsure/prefer not to answer	6 (3.3)
Race, n (%)	
White	175 (96.2)
Black	3 (1.6)
South Asian	1 (0.6)
Other	3 (1.6)
Education, n (%)	
High school or other certification	110 (60.4)
University education or higher	72 (39.6)
Household income (CAD $)^b^, n (%)	
<50,000	38 (20.9)
50,000‐99,999	71 (39.0)
≥100,000	55 (30.2)
Do not know/prefer not to answer	18 (9.9)
Employment status, n (%)	
Working full- or part-time	90 (49.5)
Retired/not working	92 (50.5)
Currently partnered, n (%)	131 (72.0)
Weight 2 years before enrollment (lb), median (IQR)	170 (145-206)
Current weight (lb), median (IQR)	194 (165-235)
BMI (kg/m^2^), median (IQR)	32 (27-38)
Obesity (BMI≥30), n (%)	107 (58.8)
Number of chronic conditions, median (IQR)	3 (1-3)
Charlson Comorbidity Index (age-adjusted), median (IQR)	2 (1-3)
Rural residence (Municipality of Pictou County), n (%)	89 (48.9)
Current smoking, n (%)	
Daily	6 (3.3)
Less than daily	2 (1.1)
Not at all	174 (95.6)
Cannabis use (past 6 months), n (%)	
Never	77 (42.3)
Former use	61 (33.5)
1‐7 times	28 (15.4)
≥8 times	16 (8.8)
Alcohol use (past 6 months), n (%)	
Lifetime abstainer	4 (2.2)
Former drinker	31 (17.0)
≤monthly	52 (28.6)
Weekly	45 (24.7)
≥3 days per week	31 (17.0)
Has a regular primary care provider, n (%)	136 (74.7)
Any doctor visit in past 12 months, n (%)	172 (94.5)
Prescribed medication for anxiety or depression, n (%)	76 (41.8)
Screened positive for psychological distress, n (%)	88 (48.4)
Physical activity level, n (%)	
Not very active	95 (52.2)
Moderately active	61 (33.5)
Very active	26 (14.3)

a Values are median (IQR) or n (%); percentages may not sum to 100 due to rounding.

b CAD $1=US $0.73 as of August 29, 2025 (Bank of Canada).

### Primary Outcomes: Longitudinal Changes in Psychological Outcomes

In adjusted population-averaged GEE models, all primary psychological outcomes improved from baseline to 6 months, with effects sustained or larger at 12 months (Table 2; Figure 2). Illness perceptions (B-IPQ) decreased at 6 months (β=−5.1, 95% CI −7.3 to −3.0; *P*<.001) and 12 months (β=−7.3, 95% CI −9.7 to −4.9; *P*<.001). Perceived stress (PSS-10) declined at 6 months (β=−2.2, 95% CI −3.2 to −1.2; *P*<.001) and 12 months (β=−2.4, 95% CI −3.5 to −1.3; *P*<.001), and disease-related worry (QSC-R10) decreased at 6 months (β=−3.7, 95% CI −5.2 to −2.3; *P*<.001) and 12 months (β=−6.3, 95% CI −7.8 to −4.7; *P*<.001). Life satisfaction (SWLS) increased at 6 months (β=1.5, 95% CI 0.5-2.6; *P*=.004) and 12 months (β=2.5, 95% CI 1.4-3.5; *P*<.001). Well-being (WHO-5) increased at 6 months (β=10.1, 95% CI 7.2-13.0; *P*<.001) and 12 months (β=10.4, 95% CI 7.2-13.5; *P*<.001). Mental health–related quality of life (SF-12 MCS) improved at 6 months (β=3.5, 95% CI 1.8-5.2; *P*<.001) and 12 months (β=4.3, 95% CI 2.4-6.2; *P*<.001).

**Table 2.** Adjusted population-averaged mean differences from baseline in continuous outcomes at 6 and 12 months (generalized estimating equation models).^a^

Outcome	β (vs baseline) at 6 months	95% CI	*P* value	β (vs baseline) at 12 months	95% CI	*P* value
B-IPQ^b^	−5.1	−7.3 to −3.0	<.001	−7.3	−9.7 to −4.9	<.001
PSS-10^c^	−2.2	−3.2 to −1.2	<.001	−2.4	−3.5 to −1.3	<.001
QSC-R10^d^	−3.7	−5.2 to −2.3	<.001	−6.3	−7.8 to −4.7	<.001
SWLS^e^	1.5	0.5 to 2.6	.004	2.5	1.4 to 3.5	<.001
WHO-5^f^	10.1	7.2 to 13.0	<.001	10.4	7.2 to 13.5	<.001
FACIT-SP-12^g^	2.9	1.8 to 4.0	<.001	4.6	3.0 to 6.2	<.001
MOS-SSS^h^	0.19	0.09 to 0.30	<.001	0.10	−0.01 to 0.21	.08
SBQ^i^	−5.7	−10.0 to −1.4	.01	−8.7	−13.2 to −4.2	<.001
FACIT-F^j^	3.6	2.0 to 5.2	<.001	5.0	3.3 to 6.8	<.001
REAP-S^k^	3.0	2.4 to 3.7	<.001	2.6	2.0 to 3.3	<.001
PSQI^l^	−1.8	−2.4 to −1.2	<.001	−2.2	−2.8 to −1.5	<.001
SF-12 PCS^m^	1.8	0.3 to 3.3	.02	2.9	1.0 to 4.9	.003
SF-12 MCS^n^	3.5	1.8 to 5.2	<.001	4.3	2.4 to 6.2	<.001
BMI	−1.2	−1.8 to −0.7	<.001	−1.7	−2.5 to −0.9	<.001
Weight (lb)	−7.2	−10.4 to −4.0	<.001	−10.3	−15.2 to −5.4	<.001
Drink score	−0.5	−0.8 to −0.2	.002	−0.6	−0.9 to −0.4	<.001
Binge score	−0.21	−0.42 to 0.00	.047	−0.03	−0.31 to 0.26	.90
Cannabis score	−0.13	−0.27 to 0.01	.06	−0.12	−0.27 to 0.02	.09

a β values represent adjusted mean differences from baseline. Models were adjusted for age, sex, comorbidity burden, relationship status, and psychotropic medication use.

b B-IPQ: Brief Illness Perception Questionnaire.

c PSS-10: Perceived Stress Scale.

d QSC-R10: Questionnaire on Distress in Cancer Patients.

e SWLS: Satisfaction With Life Scale.

f WHO-5: World Health Organization-Five Well-Being Index.

g FACIT-SP-12: Functional Assessment of Chronic Illness Therapy—Spiritual Well-Being scale.

h MOS-SSS: Medical Outcomes Study Social Support Survey.

i SBQ: Sedentary Behavior Questionnaire.

j FACIT-F: Functional Assessment of Chronic Illness Therapy—Fatigue scale.

k REAP-S: Rapid Eating Assessment for Participants—Shortened.

l PSQI: Pittsburgh Sleep Quality Index.

m SF-12 PCS: 12-item Short Form Health Survey Physical Component Summary.

n SF-12 MCS: 12-item Short Form Health Survey Mental Component Summary.

**Figure 2.**
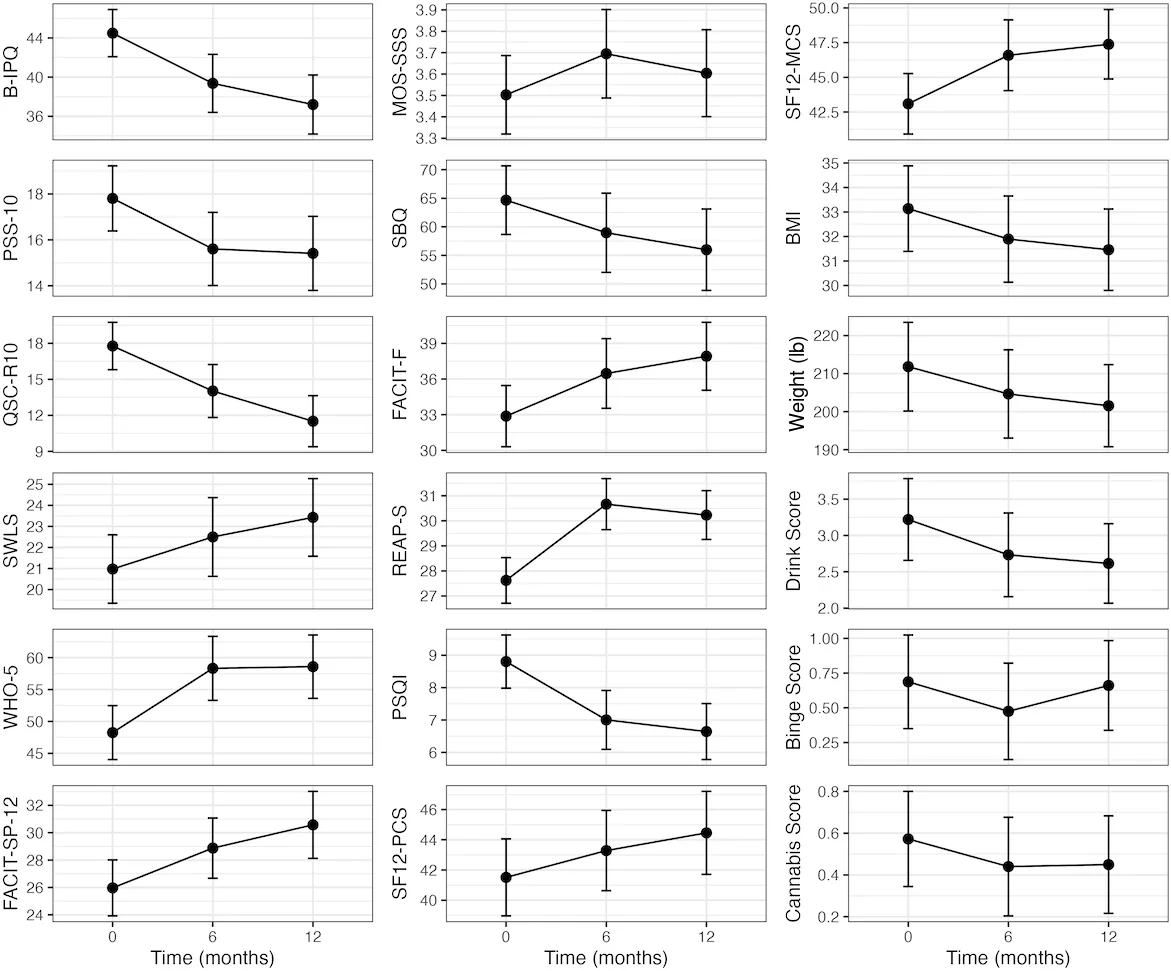
Adjusted marginal means (95% CI) at baseline (0 months), 6 months, and 12 months for continuous outcomes. Marginal means were estimated from adjusted population-averaged generalized estimating equation models including age, sex, comorbidity burden, relationship status, and baseline use of medication for depression or anxiety. Lower scores represent improvement for B-IPQ, PSS-10, QSC-R10, SBQ, PSQI, BMI, weight, and substance use frequency measures, whereas higher scores represent improvement for the positively scored measures. B-IPQ: Brief Illness Perception Questionnaire; FACIT-F: Functional Assessment of Chronic Illness Therapy—Fatigue scale; FACIT-SP-12: Functional Assessment of Chronic Illness Therapy—Spiritual Well-Being scale; MOS-SSS: Medical Outcomes Study Social Support Survey; PSQI: Pittsburgh Sleep Quality Index; PSS-10: Perceived Stress Scale; QSC-R10: Questionnaire on Distress in Cancer Patients; REAP-S: Rapid Eating Assessment for Participants—Shortened; SBQ: Sedentary Behavior Questionnaire; SF-12 MCS: 12-item Short Form Health Survey Mental Component Summary; SF-12 PCS: 12-item Short Form Health Survey Physical Component Summary; SWLS: Satisfaction With Life Scale; WHO-5: World Health Organization-Five Well-Being Index.

### Secondary Outcomes: Multidomain Health Changes

Several secondary outcomes improved over follow-up (Table 2; Figure 2). Fatigue (FACIT-F) improved at 6 months (β=3.6, 95% CI 2.0-5.2; *P*<.001) and 12 months (β=5.0, 95% CI 3.3-6.8; *P*<.001), and sleep quality (PSQI) improved at 6 months (β=−1.8, 95% CI −2.4 to −1.2; *P*<.001) and 12 months (β=−2.2, 95% CI −2.8 to −1.5; *P*<.001). Diet quality (REAP-S) improved at 6 months (β=3.0, 95% CI 2.4-3.7; *P*<.001) and 12 months (β=2.6, 95% CI 2.0-3.3; *P*<.001), and sedentary behavior (SBQ) decreased at 6 months (β=−5.7, 95% CI −10.0 to −1.4; *P*=.01) and 12 months (β=−8.7, 95% CI −13.2 to −4.2; *P*<.001). Spiritual well-being (FACIT-SP-12) improved at 6 months (β=2.9, 95% CI 1.8-4.0; *P*<.001) and 12 months (β=4.6, 95% CI 3.0-6.2; *P*<.001). Perceived social support (MOS-SSS) increased at 6 months (β=0.2, 95% CI 0.1-0.3; *P*<.001) but was not clearly different from baseline at 12 months (β=0.1, 95% CI −0.01 to 0.21; *P*=.08). Physical health–related quality of life (SF-12 PCS) improved at 6 months (β=1.8, 95% CI 0.3-3.3; *P*=.02) with a larger improvement at 12 months (β=2.9, 95% CI 1.0-4.9; *P*=.003). Weight and BMI decreased at both follow-ups (weight, 6 months: β=−7.2 lb, 95% CI −10.4 to −4.0, *P*<.001; 12 months: β=−10.3 lb, 95% CI −15.2 to −5.4, *P*<.001; BMI, 6 months: β=−1.2, 95% CI −1.8 to −0.7, *P*<.001; 12 months: β=−1.7, 95% CI −2.5 to −0.9, *P*<.001). Alcohol frequency decreased at 6 months (Drink score β=−0.5, 95% CI −0.8 to −0.2; *P*=.002) and 12 months (β=−0.6, 95% CI −0.9 to −0.4; *P*<.001). Binge drinking frequency showed a small reduction at 6 months (β=−0.21, 95% CI −0.42 to 0.00; *P*=.047) but was not clearly different from baseline at 12 months (β=−0.03, 95% CI −0.31 to 0.26; *P*=.90), and cannabis use frequency did not show a clear change at either follow-up (6 months: β=−0.13, 95% CI −0.27 to 0.01; *P*=.06; 12 months: β=−0.12, 95% CI −0.27 to 0.02; *P*=.09). Model-estimated marginal means and full contrasts for all continuous outcomes are provided in Tables S5 and S6 in Multimedia Appendix 1.

### Binary Clinical Threshold Outcomes

Relative to baseline, the odds of low well-being (WHO-5<50) were lower at 6 months (odds ratio [OR] 0.17, 95% CI 0.11-0.26; *P*<.001) and 12 months (OR 0.15, 95% CI 0.10-0.24; *P*<.001) (Table 3). The odds of high perceived stress (PSS-10≥20) were lower at 6 months (OR 0.31, 95% CI 0.21-0.48; *P*<.001) and 12 months (OR 0.20, 95% CI 0.13-0.33; *P*<.001). The odds of clinically elevated disease-related worry (QSC-R10>14) decreased at 6 months (OR 0.39, 95% CI 0.26-0.57; *P*<.001) and 12 months (OR 0.24, 95% CI 0.15-0.37; *P*<.001). Poor sleep (PSQI>5) decreased at 6 months (OR 0.45, 95% CI 0.30-0.68; *P*<.001) and 12 months (OR 0.33, 95% CI 0.21-0.52; *P*<.001), and high sedentary behavior (≥8 hours/day; ≥56 hours/week) decreased at 6 months (OR 0.59, 95% CI 0.39-0.89; *P*=.01) and 12 months (OR 0.43, 95% CI 0.28-0.65; *P*<.001). Obesity prevalence (BMI≥30) did not change at 6 months (OR 0.88, 95% CI 0.72-1.07; *P*=.20) or 12 months (OR 0.88, 95% CI 0.67-1.15; *P*=.30).

**Table 3.** Adjusted odds ratios (ORs) for meeting clinical threshold outcomes at 6 and 12 months vs baseline (GEE^a^ models^b^).

Outcome (threshold)	OR (vs baseline), 6 months	95% CI	*P* value	OR (vs baseline), 12 months	95% CI	*P* value
Low well-being (WHO-5^c^<50)	0.17	0.11-0.26	<.001	0.15	0.10-0.24	<.001
High perceived stress (PSS-10^d^≥20)	0.31	0.21-0.48	<.001	0.20	0.13-0.33	<.001
Clinically elevated worry (QSC-R10^e^>14)	0.39	0.26-0.57	<.001	0.24	0.15-0.37	<.001
Obesity (BMI≥30)	0.88	0.72-1.07	.20	0.88	0.67-1.15	.30
Poor sleep (PSQI^f^>5)	0.45	0.30-0.68	<.001	0.33	0.21-0.52	<.001
High sedentary time (SBQ^g^≥56 hours/week)	0.59	0.39-0.89	.011	0.43	0.28-0.65	<.001

a GEE: generalized estimating equation.

b ORs represent adjusted odds ratios relative to baseline. Models were adjusted for age, sex, comorbidity burden, relationship status, and psychotropic medication use.

c WHO-5: World Health Organization-Five Well-Being Index.

d PSS-10: Perceived Stress Scale.

e QSC-R10: Questionnaire on Distress in Cancer Patients.

f PSQI: Pittsburgh Sleep Quality Index.

g SBQ: Sedentary Behavior Questionnaire.

Responder analyses showed clinically meaningful improvement across multiple domains (Table S7 in Multimedia Appendix 1). At 6 and 12 months, respectively, 39 of 107 (36.4%, 95% CI 27.3%-45.6%) and 46 of 106 (43.4%, 95% CI 34.0%-52.8%) participants achieved a ≥4-point reduction in perceived stress (PSS-10), while 53 of 108 (49.1%, 95% CI 39.6%-58.5%) and 47 of 108 (43.5%, 95% CI 34.2%-52.9%) achieved a ≥10-point improvement in mental well-being (WHO-5). At 12 months, clinically meaningful improvements were also observed for illness perceptions (B-IPQ; 54/108, 50.0%, 95% CI 40.6%-59.4%), disease-related worry (QSC-R10; 56/111, 50.5%, 95% CI 41.1%-59.8%), fatigue (FACIT-F; 62/109, 56.9%, 95% CI 47.6%-66.2%), physical health–related quality of life (SF-12 PCS; 60/111, 54.1%, 95% CI 44.8%-63.3%), mental health–related quality of life (SF-12 MCS; 53/111, 47.7%, 95% CI 38.5%-57.0%), and ≥3% weight loss (52/115, 45.2%, 95% CI 36.1%-54.3%).

### Exploratory Analyses: Co-Occurring Changes in Candidate Factors and Psychological Outcomes

Exploratory adjusted models examined associations between changes in prespecified candidate factors and changes in psychological outcomes (Table 4). Improvements in fatigue were consistently associated with improvements in well-being, life satisfaction, perceived stress, and disease-related worry. From baseline to 6 months, improvement in FACIT-F scores was associated with improvement in WHO-5 (β=0.9, 95% CI 0.6 to 1.2; *P*<.001), SWLS (β=0.3, 95% CI 0.2-0.4; *P*<.001), PSS-10 (β=−0.4, 95% CI −0.5 to −0.3; *P*<.001), and QSC-R10 (β=−0.6, 95% CI −0.8 to −0.5; *P*<.001) scores.

**Table 4.** Exploratory associations between change in candidate factors and change in psychological outcomes from baseline to 6 and 12 months (adjusted GEE^a^ models).^b^

Interval and candidate factor (Δ)	ΔWHO-5^c^		ΔSWLS^d^		ΔPSS-10^e^		ΔQSC-R10^f^	
	β (95% CI)	*P* value	β (95% CI)	*P* value	β (95% CI)	*P* value	β (95% CI)	*P* value
Baseline to 6 months								
Fatigue (FACIT-F^g^)	0.9 (0.6 to 1.2)	<.001	0.3 (0.2 to 0.4)	<.001	−0.4 (−0.5 to −0.3)	<.001	−0.6 (−0.8 to −0.5)	<.001
Sleep quality (PSQI^h^)	−2.2 (−3.1 to −1.3)	<.001	−0.8 (−1.2 to −0.5)	<.001	0.8 (0.4 to 1.1)	<.001	1.4 (0.9 to 1.8)	<.001
Mental HRQoL^i^ (SF-12 MCS^j^)	0.9 (0.6 to 1.2)	<.001	0.3 (0.2 to 0.4)	<.001	−0.4 (−0.5 to −0.3)	<.001	−0.4 (−0.6 to −0.3)	<.001
Social support (MOS-SSS^k^)	8.2 (3.8 to 12.6)	<.001	3.3 (1.6 to 5.0)	<.001	−2.4 (−4.0 to −0.8)	.004	−2.4 (−4.9 to −0.02)	.049
Sedentary behavior (SBQ^l^)	−0.10 (−0.22 to 0.03)	.12	−0.06 (−0.11 to −0.02)	.005	0.05 (0.01 to 0.10)	.01	0.13 (0.07 to 0.20)	<.001
Diet quality (REAP-S^m^)	1.1 (0.2 to 1.9)	.01	0.4 (0.1 to 0.7)	.02	−0.5 (−0.8 to −0.2)	<.001	−0.8 (−1.2 to −0.4)	<.001
Spiritual well-being (FACIT-SP-12^n^)	1.1 (0.7 to 1.5)	<.001	0.5 (0.3 to 0.6)	<.001	−0.4 (−0.5 to −0.2)	<.001	−0.4 (−0.6 to −0.2)	.001
Illness perceptions (B-IPQ^o^)	−0.6 (−0.8 to −0.4)	<.001	−0.2 (−0.3 to −0.1)	<.001	0.3 (0.2 to 0.3)	<.001	0.4 (0.3 to 0.5)	<.001
Baseline to 12 months								
Fatigue (FACIT-F)	1.3 (1.0 to 1.6)	<.001	0.2 (0.1 to 0.4)	<.001	−0.4 (−0.5 to −0.3)	<.001	−0.6 (−0.7 to −0.5)	<.001
Sleep quality (PSQI)	−2.8 (−3.7 to −1.9)	<.001	−0.7 (−1.0 to −0.4)	<.001	0.7 (0.4 to 1.0)	<.001	1.4 (1.0 to 1.8)	<.001
Mental HRQoL (SF-12 MCS)	0.8 (0.5 to 1.2)	<.001	0.2 (0.1 to 0.3)	<.001	−0.3 (−0.4 to −0.2)	<.001	−0.3 (−0.5 to −0.1)	<.001
Social support (MOS-SSS)	8.5 (3.6 to 13.4)	<.001	2.7 (1.2 to 4.2)	<.001	−1.9 (−3.7 to −0.1)	.04	−4.3 (−6.6 to −2.0)	<.001
Sedentary behavior (SBQ)	−0.17 (−0.31 to −0.03)	.02	−0.06 (−0.11 to −0.02)	.008	0.03 (−0.02 to 0.08)	.30	0.12 (0.05 to 0.18)	<.001
Diet quality (REAP-S)	1.6 (0.7 to 2.6)	.001	0.4 (0.06 to 0.7)	.02	−0.4 (−0.8 to −0.1)	.02	−0.8 (−1.2 to −0.3)	.001
Spiritual well-being (FACIT-SP-12)	1.3 (1.0 to 1.6)	<.001	0.4 (0.3 to 0.5)	<.001	−0.3 (−0.4 to −0.2)	<.001	−0.5 (−0.7 to −0.3)	<.001
Illness perceptions (B-IPQ)	−0.5 (−0.8 to −0.3)	<.001	−0.2 (−0.3 to −0.1)	<.001	0.2 (0.1 to 0.3)	<.001	0.4 (0.3 to 0.5)	<.001

a GEE: generalized estimating equation.

b β values represent adjusted associations between change in the candidate mediator (Δ) and change in the psychological outcome (Δ) over the specified interval. Models were adjusted for age, sex, relationship status, Charlson Comorbidity Index, psychotropic medication use, and baseline outcome values. Analyses are exploratory and hypothesis-generating.

c WHO-5: World Health Organization-Five Well-Being Index.

d SWLS: Satisfaction With Life Scale.

e PSS-10: Perceived Stress Scale.

f QSC-R10: Questionnaire on Distress in Cancer Patients.

g FACIT-F: Functional Assessment of Chronic Illness Therapy—Fatigue scale.

h PSQI: Pittsburgh Sleep Quality Index.

i HRQoL: health-related quality of life.

j SF-12 MCS: 12-item Short Form Health Survey Mental Component Summary.

k MOS-SSS: Medical Outcomes Study Social Support Survey.

l SBQ: Sedentary Behavior Questionnaire.

m REAP-S: Rapid Eating Assessment for Participants—Shortened.

n FACIT-SP-12: Functional Assessment of Chronic Illness Therapy—Spiritual Well-Being scale.

o B-IPQ: Brief Illness Perception Questionnaire.

Improvements in sleep quality were similarly associated with improvements across psychological outcomes. From baseline to 6 months, improvement in PSQI scores was associated with improvement in WHO-5 (β=−2.2, 95% CI −3.1 to −1.3; *P*<.001), SWLS (β=−0.8, 95% CI −1.2 to −0.5; *P*<.001), PSS-10 (β=0.8, 95% CI 0.4 to 1.1; *P*<.001), and QSC-R10 (β=1.4, 95% CI 0.9 to 1.8; *P*<.001).

More favorable illness perceptions were also associated with improvements across psychological outcomes. From baseline to 6 months, improvement in B-IPQ scores was associated with improvement in WHO-5 (β=−0.6, 95% CI −0.8 to −0.4; *P*<.001), SWLS (β=−0.2, 95% CI −0.3 to −0.1; *P*<.001), PSS-10 (β=0.3, 95% CI 0.2-0.3; *P*<.001), and QSC-R10 (β=0.4, 95% CI 0.3-0.5; *P*<.001). Similar patterns of association were observed at 12 months (Table 4). Additional exploratory associations between changes in candidate factors and psychological outcomes are reported in Tables S8 and S9 in Multimedia Appendix 1.

### Exploratory Analyses: Moderators of Psychological Trajectories

Exploratory moderation analyses assessed whether baseline characteristics modified trajectories of psychological change (Table 5). Higher baseline perceived stress was associated with larger reductions in perceived stress at 12 months (time × baseline PSS-10: β=−0.4, 95% CI −0.6 to −0.3; *P*<.001) and greater improvement in well-being (time × baseline PSS-10 for WHO-5: β=0.6, 95% CI 0.2-1.1; *P*=.007). Higher baseline well-being was associated with smaller improvement in well-being (time × baseline WHO-5: β=−0.2, 95% CI −0.3 to −0.1; *P*<.001), and higher baseline life satisfaction was associated with smaller gains in life satisfaction (time × baseline SWLS: β=−0.4, 95% CI −0.5 to −0.2; *P*<.001). Baseline illness perceptions did not modify trajectories of well-being (time × baseline B-IPQ: β=0.13, 95% CI −0.10 to 0.37; *P*=.30) or perceived stress (time × baseline B-IPQ for PSS-10: β=−0.06, 95% CI −0.15 to 0.04; *P*=.20). Full interaction outputs are provided in Table S10 in Multimedia Appendix 1.

**Table 5.** Baseline psychological moderators of longitudinal change in psychological outcomes (baseline to 12 months).^a^

Baseline psychological characteristic	Outcome	Direction of moderation	Interaction β (95% CI)	*P* value
Perceived stress (PSS-10^b^)	WHO-5^c^	Higher baseline stress associated with greater improvement in well-being	0.6 (0.2 to 1.1)	.007
	PSS-10	Higher baseline stress associated with larger reductions in perceived stress	−0.4 (−0.6 to −0.3)	<.001
Illness perceptions (B-IPQ^d^)	WHO-5	No evidence of effect modification	0.13 (−0.10 to 0.37)	.30
	PSS-10	No evidence of effect modification	−0.06 (−0.15 to 0.04)	.20
Psychological well-being (WHO-5)	WHO-5	Higher baseline well-being associated with smaller gains	−0.2 (−0.3 to −0.1)	<.001
Life satisfaction (SWLS^e^)	SWLS	Higher baseline life satisfaction associated with smaller gains	−0.4 (−0.5 to −0.2)	<.001

a β coefficients represent time × baseline characteristic interaction terms from adjusted generalized estimating equation models, reflecting differential change from baseline to 12 months. Models were adjusted for age, sex, relationship status, Charlson Comorbidity Index, and psychotropic medication use. Only prespecified psychological moderators with stable and interpretable interaction effects are shown.

b PSS-10: Perceived Stress Scale.

c WHO-5: World Health Organization-Five Well-Being Index.

d B-IPQ: Brief Illness Perception Questionnaire.

e SWLS: Satisfaction With Life Scale.

## Discussion

### Principal Findings

This secondary analysis of PictouCountyPEP found sustained improvements across multiple psychological, behavioral, and physical health domains over 12 months in rural adults living with chronic conditions. Improvements were observed across all primary psychological outcomes and were accompanied by favorable changes in fatigue, sleep, diet quality, sedentary behavior, spiritual well-being, and mental health–related quality of life. Exploratory analyses further suggested that psychological improvements co-occurred with changes in fatigue, sleep, illness perceptions, social support, and spiritual well-being, pointing to plausible multidomain pathways through which digital self-management interventions may influence well-being over time.

Exploratory analyses further showed that improvements in several candidate factors, notably fatigue, sleep quality, illness perceptions, social support, and spiritual well-being, were associated with improvements in psychological outcomes. These patterns of co-occurring improvement may help identify candidate multidomain pathways for future investigation but should not be interpreted as evidence of statistical mediation or causal mechanisms. Establishing whether these factors mediate intervention-related psychological outcomes will require an appropriate temporal and causal analytic framework, ideally within a controlled study designed prospectively to test these pathways.

Social support and binge drinking frequency were less clearly improved at 12 months, and improvements in diet quality were somewhat attenuated, but the overall pattern remained favorable. Binary threshold and responder analyses were broadly concordant with the continuous outcome models. Obesity status was the main exception: although the odds of meeting the binary obesity threshold did not change, continuous BMI and weight outcomes improved, as did responder analyses based on clinically meaningful weight loss. Program engagement, adherence, and acceptability have been reported elsewhere and are therefore not repeated here [20].

These findings contribute to the growing literature on digital health interventions for chronic disease self-management in community settings [53]. Importantly, this study evaluated the program under real-world delivery conditions rather than within a tightly controlled clinical trial environment. Participants accessed the intervention remotely using their own devices, engagement occurred asynchronously, and optional components were used flexibly. These characteristics provide context for interpreting the observed outcomes and for future evaluation of feasibility, accessibility, and acceptability in routine community settings [53]. Such implementation-oriented evidence is particularly relevant for digital health interventions intended to reach geographically dispersed populations [20,53].

The sustained psychological improvements observed at 12 months contrast with several recent systematic reviews and meta-analyses of digital interventions, which commonly report attenuation of mental health effects over time. For example, digital behavioral activation interventions have shown reductions in depressive symptoms at shorter follow-up, but no significant pooled benefit at 12 months; pooled anxiety outcomes were not significantly improved at the assessed follow-up points [54]. Digital peer support interventions have similarly demonstrated weaker mental health effects with longer follow-up [55], and digital exercise interventions have shown improvements at the end of the intervention that are not consistently maintained at later follow-up [56]. By contrast, psychological improvements in PictouCountyPEP remained evident at 12 months, during the lower-intensity maintenance period. Although direct comparisons across interventions and follow-up designs should be made cautiously, these findings suggest that comprehensive, multicomponent approaches warrant further evaluation for their potential to support longer-term psychological benefits. These findings complement evidence that interventions targeting multiple health behaviors can support chronic disease management [11] and indicate a need to evaluate their longer-term psychological outcomes.

The durability of effects may also reflect intervention design choices intended to reduce common barriers to digital engagement in rural settings, including low-bandwidth delivery, brief daily content, and minimal onboarding requirements [57]. Although engagement mechanisms were not directly tested in the present study, this microdose format may support habit formation and continued participation among individuals managing fluctuating symptoms and competing life demands [58]. These findings are also relevant to the well-described mismatch between health care systems historically oriented toward acute, reactive care and the proactive, ongoing support required for effective chronic disease self-management [59,60]. Many determinants most closely linked to symptom burden and progression unfold between clinical encounters, including sleep disruption, fatigue, stress, dietary patterns, social connection, and illness perceptions [60]. In this context, PictouCountyPEP may function as a low-burden adjunct that supports daily self-regulation and patient capability in routine living, complementing rather than replacing biomedical care.

Exploratory moderation analyses addressed an important question for digital self-management programs: for whom do these interventions appear to work best? Participants with higher baseline perceived stress experienced larger reductions in stress and greater improvements in well-being over 12 months, whereas those with higher baseline well-being or life satisfaction showed smaller gains in those same domains. This pattern suggests that the program may confer greater benefit among individuals with greater baseline psychological need. We found no consistent evidence that demographic characteristics substantially altered psychological trajectories, although these exploratory analyses may have been underpowered to detect smaller subgroup differences.

The exploratory mechanistic analyses point to several plausible pathways through which psychological improvement may occur. One pathway may involve modification of upstream cognitive processes, including illness perceptions that shape coping and behavioral responses, consistent with Leventhal’s common-sense model of self-regulation [61]. Another may reflect increased engagement in meaningful, reinforced daily activities supported by social connection and sustained routines, aligning with behavioral activation frameworks [62]. More broadly, the observed pattern is consistent with interdependent changes across energy, sleep, cognition, and social resources that collectively support psychological recovery [61-63]. These findings extend earlier work on Prostate Cancer Patient Empowerment Program (PC-PEP), in which self-efficacy and illness perceptions explained improvements in psychological distress [64]. However, these analyses are exploratory and should not be interpreted as evidence of causal mediation. Future studies should formally test these candidate pathways using longitudinal mediation models or experimental designs.

Although this study was not designed to assess disease incidence, the improvements observed across stress, sleep, diet quality, sedentary behavior, and social support are relevant to secondary prevention in chronic disease. These domains represent potentially modifiable targets relevant to chronic disease self-management and health [60,65]. Future controlled studies with longer follow-up should examine whether these changes translate into downstream clinical outcomes, multimorbidity accumulation, or health service use.

Beyond the observed health improvements, PictouCountyPEP builds on a digital delivery model already implemented in PC-PEP across Canada and internationally. The program delivers core content through automated email prompts and brief asynchronous video modules, limiting the need for clinicians to deliver individual sessions. An international phase 4 implementation trial of PC-PEP reported similar follow-up completion across rural and urban participants, high program acceptability, and sustained improvements in mental health [66]. This implementation experience supports the scalability of the delivery model and provides a foundation for extending it to rural chronic disease self-management. Broader implementation of PictouCountyPEP should build on this experience while accounting for local digital access, staffing arrangements, and integration with existing services [57,67].

From a health system perspective, scalable self-management programs that can be delivered at low cost and without reliance on specialized personnel are of increasing interest, particularly in rural and resource-constrained settings [66,68]. An economic evaluation within the PC-PEP randomized controlled trial estimated program delivery costs of CAD $200.07 (CAD $1=US $0.73 as of August 29, 2025) per participant and found that early delivery improved outcomes while lowering overall health care costs [68]. These findings provide an economic rationale for broader implementation of the delivery model. A formal cost-effectiveness analysis of PictouCountyPEP from the health care system perspective is currently underway to assess downstream resource use and longer-term value. These findings also have implications for how digital self-management programs could be integrated into chronic disease care pathways. In many health systems, patients with multimorbidity receive episodic clinical care but limited ongoing behavioral or psychosocial support between visits [59,60]. Digital empowerment programs may complement traditional care by providing continuous, low-intensity behavioral guidance that patients can access independently. For primary care teams serving rural populations, such programs could function as an adjunct resource that reinforces lifestyle recommendations, supports stress regulation, and promotes patient activation without substantially increasing clinical workload. Work is currently underway in collaboration with Nova Scotia Health to explore potential implementation pathways for the program within existing care structures. Together with the ongoing cost-effectiveness analysis, these efforts aim to determine whether digitally delivered self-management programs can offer a sustainable and cost-efficient complement to traditional chronic disease care models.

### Nursing and Primary Care Implications

Although PictouCountyPEP was not designed as a nurse-delivered intervention, its asynchronous, home-based structure has potential implications for nursing and primary care practice in rural settings. Nurse-led remote digital approaches have been used to support adults with chronic conditions through health education, remote support, monitoring, and other forms of digitally enabled care [69]. More broadly, digital interventions are increasingly being evaluated as adjuncts to chronic disease self-management, although their effectiveness varies across conditions, intervention components, and self-care domains [65]. Rather than requiring nurses to deliver the intervention content directly, a program of this type could provide a standardized adjunct to health education and self-management support, with nurses and other primary care professionals potentially identifying appropriate patients, reinforcing individualized self-management goals, recognizing emerging needs during routine encounters, and directing patients to additional clinical or community services when required. This model may be particularly relevant in settings where clinical time and access to specialized chronic disease services are constrained because the core educational and behavioral content is delivered outside the clinical encounter and does not require continuous clinician involvement [69].

These implementation questions are particularly relevant in the present rural context: at baseline, 19 of 182 (10.4%) participants reported receiving primary care from another provider such as a nurse practitioner, while 46 of 182 (25.3%) participants reported having no regular primary care provider (Table S3 in Multimedia Appendix 1). Importantly, however, these potential nursing and primary care roles were not evaluated in the present study. Future implementation research should examine how referral to digital self-management programs can be incorporated into rural primary care and nursing workflows, including which patients are most appropriate for referral, whether some patients require additional adherence or digital navigation support, and whether integration can extend self-management support without increasing clinical workload.

### Limitations

This study has several limitations. First, the study was conducted in a single, predominantly rural region, and the sample was predominantly female (85.2%) and White (96.2%), limiting generalizability to men and more ethnoculturally diverse populations. Eligibility also required English proficiency and access to the internet and email; consequently, the findings cannot be assumed to generalize to non–English-speaking populations or to individuals experiencing greater barriers to digital access or with lower digital health literacy. Although the intervention was intentionally designed for low-bandwidth delivery and did not require specialized applications, wearable devices, or synchronous participation, these design features do not eliminate potential digital access or digital literacy barriers. Generalizability to other rural regions may also depend on local sociodemographic characteristics, health care access, digital infrastructure, and community resources [57,67]. Similarly, the findings should not be assumed to apply directly to urban populations, where health service availability, referral pathways, and opportunities for in-person chronic disease support may differ. Evaluation in more diverse populations and geographic settings is therefore needed. Second, the single-arm design precludes causal attribution of the observed longitudinal changes to PictouCountyPEP. Without a concurrent control group, changes associated with natural disease trajectories, secular or time effects, regression to the mean, unmeasured co-interventions, and residual confounding cannot be separated from changes occurring during program participation. Although covariate adjustment and sensitivity analyses strengthen the assessment of longitudinal associations, they do not overcome this design limitation. Future randomized controlled trials should evaluate the efficacy of PictouCountyPEP relative to an appropriate comparator and determine whether the observed multidomain changes are attributable to the intervention. Third, all outcomes were self-reported and may therefore be subject to recall, reporting, and social desirability biases. The study also did not assess longer-term clinical or health service outcomes, such as hospitalization or mortality; consequently, the observed changes in patient-reported outcomes should not be interpreted as evidence of effects on these outcomes. Fourth, participants with incomplete follow-up had lower baseline well-being and life satisfaction, raising the possibility of attrition-related bias. However, none of the included variables was significantly associated with incomplete follow-up in the adjusted model, and the primary findings were consistent in complete case and weighted sensitivity analyses. Fifth, because multiple outcomes and exploratory analyses were examined without formal adjustment for multiple comparisons, some statistically significant findings may reflect chance. Accordingly, secondary and exploratory findings should be interpreted with caution, with emphasis on consistency and effect size rather than statistical significance alone. Finally, responder analyses were only possible for measures with available thresholds for meaningful change; for some outcomes, such thresholds remain poorly defined in general chronic disease populations. Future research should prioritize the development and validation of clinically meaningful benchmarks across relevant outcomes in chronic disease settings. Notwithstanding these limitations, strengths of the study include its comprehensive multidomain scope, use of validated instruments, prospective longitudinal design, and analytic approach incorporating all available observed outcome data, supported by complete case and inverse probability–weighted sensitivity analyses.

### Conclusion

PictouCountyPEP is a digitally delivered self-management program associated with sustained improvements across multiple psychological, behavioral, and physical health domains in rural adults living with chronic conditions. These findings suggest that low-burden, multicomponent digital interventions may support sustained improvements in mental well-being alongside favorable changes in related behavioral and psychosocial outcomes. The program’s low-bandwidth, asynchronous delivery model highlights its potential scalability and relevance for rural and underserved populations, where access to ongoing behavioral and psychosocial support is limited. By addressing shared, upstream determinants of chronic disease across conditions, digitally delivered self-management programs may complement traditional care by extending support into patients’ daily lives without increasing clinical workload. Future research should evaluate long-term clinical and health system outcomes, including health service use and cost-effectiveness, and prospectively test the candidate pathways suggested by these exploratory analyses to inform intervention optimization and implementation at scale.

## Supplementary material

10.2196/95283 Multimedia Appendix 1 Tables depicting the internal consistency of instruments at baseline, baseline characteristics by follow-up status, additional baseline characteristics, follow-up status for each outcome at 6 and 12 months, model-estimated marginal means by time point for continuous outcomes, longitudinal change from baseline, results of responder analyses, associations between changes in candidate factors and changes in outcomes from baseline to 6 months and 12 months, and effect modification by baseline psychological characteristics.

## Abbreviations

B-IPQ: Brief Illness Perception Questionnaire
FACIT-F: Functional Assessment of Chronic Illness Therapy—Fatigue scale
FACIT-SP-12: 12-item Functional Assessment of Chronic Illness Therapy—Spiritual Well-Being scale
GEE: generalized estimating equation
MCS: Mental Component Summary (of the 12-item Short Form Health Survey)
MOS-SSS: Medical Outcomes Study Social Support Survey
PAR-Q+: Physical Activity Readiness Questionnaire for Everyone
PC-PEP: Prostate Cancer Patient Empowerment Program
PCS: Physical Component Summary (of the 12-item Short Form Health Survey)
PictouCountyPEP: Pictou County Patient Empowerment Program
PIPEDA: Personal Information Protection and Electronic Documents Act
PSQI: Pittsburgh Sleep Quality Index
PSS-10: 10-item Perceived Stress Scale
QSC-R10: 10-item Questionnaire on Distress in Cancer Patients
REAP-S: Rapid Eating Assessment for Participants—Shortened version
REB: Research Ethics Board
SBQ: Sedentary Behavior Questionnaire
SF-12: 12-item Short Form Health Survey
SWLS: Satisfaction With Life Scale
TREND: Transparent Reporting of Evaluations with Nonrandomized Designs
WHO-5: World Health Organization-Five Well-Being Index
