# Supplementary material for: Multidomain Outcomes of a Digital Self-Management Program in Rural Adults With Chronic Conditions: Single-Arm Prospective Interventional Study
Source: JMIR Mhealth Uhealth. 2026 Oct 30;14:e95283. doi: 10.2196/95283 (PMC13722102; doi:10.2196/95283)
Supplement: Supplementary file 1 [file mhealth-v14-e95283-s001.docx]

**Supplementary Materials for Smith et al., Longitudinal Multidomain Change in PictouCountyPEP**

[Table S1. Internal consistency of multi-item instruments at baseline	2](#_Toc224829037)

[Table S2. Baseline characteristics by follow-up status	3](#_Toc224829038)

[Table S3. Additional baseline sociodemographic, clinical, and healthcare utilisation characteristics (N = 182)	4](#_Toc224829039)

[Table S4. Follow-up status for each outcome at 6 and 12 months	5](#_Toc224829040)

[Table S5. Model-estimated marginal means by time point for continuous outcomes	6](#_Toc224829041)

[Table S6. Longitudinal change from baseline: adjusted contrasts for continuous outcomes and odds ratios for binary thresholds	7](#_Toc224829042)

[Table S7. Responder analyses at 6 and 12 months using pre-specified thresholds	8](#_Toc224829043)

[Table S8. Associations Between Changes in Candidate Factors and Changes in Outcomes From Baseline to 6 Months	9](#_Toc224829044)

[Table S9. Associations Between Changes in Candidate Factors and Changes in Outcomes From Baseline to 12 Months	10](#_Toc224829045)

[Table S10. Effect modification by baseline psychological characteristics: time × moderator interactions (exploratory)	11](#_Toc224829046)

**Table S1. Internal consistency of multi-item instruments at baseline**

| **Measure** | **N** | **Cronbach’s α** | **95% CI** |
| --- | --- | --- | --- |
| B-IPQ | 181 | 0.68 | 0.61 to 0.75 |
| PSS-10 | 182 | 0.90 | 0.88 to 0.92 |
| QSC-R10 | 182 | 0.86 | 0.83 to 0.89 |
| SWLS | 182 | 0.93 | 0.92 to 0.95 |
| WHO-5 | 182 | 0.87 | 0.83 to 0.90 |
| FACIT-F | 182 | 0.94 | 0.93 to 0.95 |
| REAP-S | 182 | 0.71 | 0.64 to 0.77 |
| PSQI | 180 | 0.72 | 0.65 to 0.77 |
| MOS-SSS | 182 | 0.97 | 0.96 to 0.97 |
| FACIT-SP-12 | 182 | 0.91 | 0.88 to 0.92 |

**Notes:** Cronbach’s α estimated at baseline. Abbreviations: B-IPQ, Brief Illness Perception Questionnaire; CI, confidence interval; FACIT-F, Functional Assessment of Chronic Illness Therapy–Fatigue; FACIT-SP-12, Functional Assessment of Chronic Illness Therapy–Spiritual Well-Being Scale; MOS-SSS, Medical Outcomes Study Social Support Survey; PSQI, Pittsburgh Sleep Quality Index; PSS-10, Perceived Stress Scale; QSC-R10, Questionnaire on Stress in Cancer; REAP-S, Rapid Eating Assessment for Participants–Shortened; SWLS, Satisfaction With Life Scale; WHO-5, World Health Organization 5-item Well-Being Index.

**Table S2. Baseline characteristics by follow-up status**

| **Characteristic** | **Incomplete Follow-up**  N = 73 | **Complete Follow-up**  N = 109 | ***P* value** |
| --- | --- | --- | --- |
| Age | 57 (45, 69) | 62 (52, 68) | 0.2 |
| Female | 59 (81%) | 96 (88%) | 0.2 |
| Charlson Comorbidity Index | 2 (1, 3) | 2 (1, 3) | 0.3 |
| Relationship Status - Partnered | 51 (70%) | 80 (73%) | 0.6 |
| Medication for Depression/Anxiety | 36 (49%) | 40 (37%) | 0.091 |
| B-IPQ | 48 (38, 53) | 43 (34, 51) | 0.12 |
| PSS-10 | 19 (12, 23) | 16 (13, 23) | 0.4 |
| QSC-R10 | 20 (11, 26) | 16 (8, 24) | 0.10 |
| SWLS | 20 (14, 29) | 26 (17, 30) | **0.013** |
| WHO-5 | 40 (24, 60) | 52 (36, 68) | **0.029** |
| **Notes:** Values are Median (Q1, Q3) for continuous variables and n (%) for categorial. *P* values are derived from Wilcoxon rank sum and Pearson's Chi-squared tests as appropriate. Complete follow-up is defined as completing assessments at all three timepoints (baseline, 6, and 12 months); numbers slightly differ from Figure 1 due to non-monotonic follow-up (e.g., some individuals missed the 6-month follow-up but completed the 12-month). Baseline data complete across all variables except B-IPQ, with 1 missing value in the incomplete follow-up group (n=72). Abbreviations: B-IPQ, Brief Illness Perception Questionnaire; PSS-10, Perceived Stress Scale; QSC-R10, Questionnaire on Stress in Cancer; SWLS, Satisfaction With Life Scale; WHO-5, World Health Organization 5-item Well-Being Index*.* | | | |

**Table S3. Additional baseline sociodemographic, clinical, and healthcare utilisation characteristics (N = 182)**

| **Characteristic** | **n (%)** |
| --- | --- |
| **Community of residence** |  |
| New Glasgow | 45 (25%) |
| Stellarton | 13 (7%) |
| Pictou | 12 (7%) |
| Westville | 16 (9%) |
| Trenton | 7 (4%) |
| Municipality of Pictou County | 89 (49%) |
| **Primary chronic condition** |  |
| Obesity / overweight | 46 (25%) |
| Diabetes (type 2) | 23 (13%) |
| Hypertension | 17 (9%) |
| Anxiety or stress disorders | 15 (8%) |
| Arthritis | 14 (8%) |
| Depression | 8 (4%) |
| Sleep disorders | 4 (2%) |
| Other conditions* | 55 (30%) |
| **Healthcare access** |  |
| Has a family doctor | 117 (64%) |
| Has another primary care provider (e.g., nurse practitioner) | 19 (10%) |
| No regular primary care provider | 46 (26%) |
| **Healthcare utilization (past 12 months)** |  |
| Any visit to a doctor or healthcare provider | 172 (95%) |
| At least one visit for physical illness | 143 (79%) |
| At least one visit for mental health | 48 (26%) |
| At least one emergency department visit (not admitted overnight) | 43 (24%) |
| At least one overnight hospitalization | 14 (8%) |
| **Binge drinking (past 6 months)** |  |
| Never | 150 (82%) |
| Any binge drinking | 32 (18%) |
| **Physical activity at work or leisure** |  |
| Not very active | 95 (52%) |
| Moderately active | 61 (34%) |
| Very active | 26 (14%) |

**Notes:** Community categories reflect municipal boundaries within Pictou County, Nova Scotia. *Other conditions include cardiovascular, respiratory, neurological, endocrine, pain, post–COVID-19, and less prevalent chronic conditions.

**Table S4. Follow-up status for each outcome at 6 and 12 months**

| **Characteristic** | **Baseline**  **n (%)** | **6 Months**  **n (%)** | **12 Months**  **n (%)** |
| --- | --- | --- | --- |
| B-IPQ | 181 (99.5%) | 110 (60.4%) | 109 (59.9%) |
| PSS-10 | 182 (100%) | 107 (58.8%) | 106 (58.2%) |
| QSC-R10 | 182 (100%) | 114 (62.6%) | 111 (61.0%) |
| SWLS | 182 (100%) | 110 (60.4%) | 108 (59.3%) |
| WHO-5 | 182 (100%) | 108 (59.3%) | 108 (59.3%) |
| FACIT-SP-12 | 182 (100%) | 114 (62.6%) | 108 (59.3%) |
| MOS-SSS | 182 (100%) | 115 (63.2%) | 107 (58.8%) |
| SBQ | 182 (100%) | 111 (61.0%) | 110 (60.4%) |
| FACIT-F | 182 (100%) | 108 (59.3%) | 109 (59.9%) |
| REAP-S | 182 (100%) | 114 (62.6%) | 114 (62.6%) |
| PSQI | 180 (98.9%) | 113 (62.1%) | 107 (58.8%) |
| SF12-PCS | 182 (100%) | 114 (62.6%) | 111 (61.0%) |
| SF12-MCS | 182 (100%) | 114 (62.6%) | 111 (61.0%) |
| BMI | 182 (100%) | 116 (63.7%) | 115 (63.2%) |
| Weight (lbs) | 182 (100%) | 119 (65.4%) | 115 (63.2%) |
| Drink Score | 182 (100%) | 115 (63.2%) | 115 (63.2%) |
| Binge Score | 182 (100%) | 115 (63.2%) | 115 (63.2%) |
| Cannabis Score | 182 (100%) | 113 (62.1%) | 115 (63.2%) |

**Notes:** Percentages are calculated out of a total possible response from 182 individuals. Abbreviations: B-IPQ, Brief Illness Perception Questionnaire; BMI, body mass index; FACIT-F, Functional Assessment of Chronic Illness Therapy–Fatigue; FACIT-SP-12, Functional Assessment of Chronic Illness Therapy–Spiritual Well-Being Scale; MOS-SSS, Medical Outcomes Study Social Support Survey; PSQI, Pittsburgh Sleep Quality Index; PSS-10, Perceived Stress Scale; QSC-R10, Questionnaire on Stress in Cancer; REAP-S, Rapid Eating Assessment for Participants–Shortened; SBQ, Sedentary Behaviour Questionnaire; SF-12 PCS/MCS, 12-item Short Form Health Survey Physical/Mental Component Summary; SWLS, Satisfaction With Life Scale; WHO-5, World Health Organization 5-item Well-Being Index.

**Table S5. Model-estimated marginal means by time point for continuous outcomes**

| **Outcome** | **Baseline mean**  **(95% CI)** | **6 months mean**  **(95% CI)** | **12 months mean**  **(95% CI)** |
| --- | --- | --- | --- |
| B-IPQ | 44.49 (42.08 to 46.90) | 39.36 (36.39 to 42.32) | 37.20 (34.18 to 40.22) |
| PSS-10 | 17.81 (16.39 to 19.22) | 15.61 (14.01 to 17.20) | 15.41 (13.80 to 17.03) |
| QSC-R10 | 17.77 (15.79 to 19.75) | 14.02 (11.82 to 16.23) | 11.50 (9.38 to 13.63) |
| SWLS | 20.98 (19.35 to 22.60) | 22.50 (20.63 to 24.37) | 23.43 (21.59 to 25.27) |
| WHO-5 | 48.25 (44.02 to 52.47) | 58.33 (53.31 to 63.35) | 58.60 (53.63 to 63.56) |
| FACIT-SP-12 | 25.97 (23.92 to 28.01) | 28.88 (26.68 to 31.07) | 30.57 (28.13 to 33.02) |
| MOS-SSS | 3.50 (3.32 to 3.69) | 3.69 (3.49 to 3.90) | 3.60 (3.40 to 3.81) |
| SBQ | 64.67 (58.66 to 70.67) | 58.96 (52.03 to 65.90) | 55.99 (48.86 to 63.12) |
| FACIT-F | 32.88 (30.30 to 35.45) | 36.47 (33.55 to 39.39) | 37.92 (35.06 to 40.78) |
| REAP-S | 27.62 (26.71 to 28.53) | 30.67 (29.65 to 31.69) | 30.23 (29.26 to 31.20) |
| PSQI | 8.80 (7.98 to 9.62) | 7.00 (6.10 to 7.91) | 6.64 (5.78 to 7.51) |
| SF-12 PCS | 41.51 (38.96 to 44.06) | 43.28 (40.63 to 45.94) | 44.46 (41.71 to 47.20) |
| SF-12 MCS | 43.09 (40.91 to 45.27) | 46.59 (44.04 to 49.14) | 47.38 (44.88 to 49.89) |
| BMI | 33.14 (31.39 to 34.88) | 31.90 (30.14 to 33.65) | 31.46 (29.80 to 33.12) |
| Weight (lbs) | 211.84 (200.17 to 223.50) | 204.66 (193.06 to 216.25) | 201.56 (190.77 to 212.35) |
| Drink Score | 3.22 (2.66 to 3.78) | 2.73 (2.16 to 3.31) | 2.61 (2.07 to 3.16) |
| Binge Score | 0.69 (0.35 to 1.02) | 0.47 (0.13 to 0.82) | 0.66 (0.34 to 0.98) |
| Cannabis Score | 0.57 (0.34 to 0.80) | 0.44 (0.20 to 0.68) | 0.45 (0.22 to 0.68) |

**Notes:** Marginal means derived from adjusted generalized estimating equation models. All models adjusted for age, sex, comorbidity, relationship status, and medication for depression/anxiety. Abbreviations: B-IPQ, Brief Illness Perception Questionnaire; BMI, body mass index; CI, confidence interval; FACIT-F, Functional Assessment of Chronic Illness Therapy–Fatigue; FACIT-SP-12, Functional Assessment of Chronic Illness Therapy–Spiritual Well-Being Scale; GEE, generalized estimating equations; MOS-SSS, Medical Outcomes Study Social Support Survey; PSQI, Pittsburgh Sleep Quality Index; PSS-10, Perceived Stress Scale; QSC-R10, Questionnaire on Stress in Cancer; REAP-S, Rapid Eating Assessment for Participants–Shortened; SBQ, Sedentary Behaviour Questionnaire; SF-12 PCS/MCS, 12-item Short Form Health Survey Physical/Mental Component Summary; SWLS, Satisfaction With Life Scale; WHO-5, World Health Organization 5-item Well-Being Index.

**Table S6. Longitudinal change from baseline: adjusted contrasts for continuous outcomes and odds ratios for binary thresholds**

**A) Continuous outcomes: adjusted mean differences from baseline**

| **Outcome** | **6 months vs baseline, adjusted mean difference (95% CI)** | ***P* value** | **12 months vs baseline, adjusted mean difference (95% CI)** | ***P* value** |
| --- | --- | --- | --- | --- |
| B-IPQ | −5.13 (−7.68 to −2.58) | <.001 | −7.29 (−10.19 to −4.39) | <.001 |
| PSS-10 | −2.20 (−3.38 to −1.02) | <.001 | −2.39 (−3.76 to −1.03) | <.001 |
| QSC-R10 | −3.75 (−5.51 to −1.98) | <.001 | −6.27 (−8.14 to −4.39) | <.001 |
| SWLS | 1.52 (0.26 to 2.78) | .013 | 2.45 (1.15 to 3.75) | <.001 |
| WHO-5 | 10.08 (6.56 to 13.60) | <.001 | 10.35 (6.53 to 14.17) | <.001 |
| FACIT-SP-12 | 2.91 (1.55 to 4.27) | <.001 | 4.61 (2.71 to 6.51) | <.001 |
| MOS-SSS | 0.19 (0.07 to 0.32) | .001 | 0.10 (−0.03 to 0.24) | .182 |
| SBQ | −5.70 (−10.90 to −0.51) | .027 | −8.68 (−14.08 to −3.27) | .001 |
| FACIT-F | 3.59 (1.62 to 5.56) | <.001 | 5.04 (2.97 to 7.12) | <.001 |
| REAP-S | 3.04 (2.27 to 3.81) | <.001 | 2.61 (1.83 to 3.39) | <.001 |
| PSQI | −1.80 (−2.52 to −1.08) | <.001 | −2.16 (−2.98 to −1.34) | <.001 |
| SF-12 PCS | 1.77 (−0.04 to 3.59) | .057 | 2.95 (0.59 to 5.30) | .010 |
| SF-12 MCS | 3.50 (1.50 to 5.49) | <.001 | 4.29 (2.01 to 6.57) | <.001 |
| BMI | −1.24 (−1.88 to −0.61) | <.001 | −1.68 (−2.63 to −0.73) | <.001 |
| Weight | −7.18 (−11.03 to −3.33) | <.001 | −10.28 (−16.17 to −4.38) | <.001 |
| Drink score | −0.49 (−0.85 to −0.12) | .005 | −0.61 (−0.90 to −0.31) | <.001 |
| Binge score | −0.21 (−0.46 to 0.04) | .118 | −0.03 (−0.37 to 0.32) | .982 |
| Cannabis score | −0.13 (−0.30 to 0.03) | .148 | −0.12 (−0.29 to 0.05) | .214 |

**Notes:** Adjusted mean differences in Table S5 were derived from marginal contrasts of model-estimated means; minor differences from Table 2 may occur due to post-estimation contrast calculation and rounding. All models adjusted for age, sex, comorbidity, relationship status, and medication for depression/anxiety. Abbreviations: B-IPQ, Brief Illness Perception Questionnaire; BMI, body mass index; CI, confidence interval; FACIT-F, Functional Assessment of Chronic Illness Therapy–Fatigue; FACIT-SP-12, Functional Assessment of Chronic Illness Therapy–Spiritual Well-Being Scale; GEE, generalized estimating equations; MOS-SSS, Medical Outcomes Study Social Support Survey; PSQI, Pittsburgh Sleep Quality Index; PSS-10, Perceived Stress Scale; QSC-R10, Questionnaire on Stress in Cancer; REAP-S, Rapid Eating Assessment for Participants–Shortened; SBQ, Sedentary Behaviour Questionnaire; SF-12 PCS/MCS, 12-item Short Form Health Survey Physical/Mental Component Summary; SWLS, Satisfaction With Life Scale; WHO-5, World Health Organization 5-item Well-Being Index.

**B) Binary clinical threshold outcomes: adjusted odds ratios relative to baseline**

| **Binary outcome** | **6 months vs baseline, adjusted OR (95% CI)** | ***P* value** | **12 months vs baseline, adjusted OR (95% CI)** | ***P* value** |
| --- | --- | --- | --- | --- |
| WHO-5 low (<50) | 0.17 (0.10 to 0.28) | <.001 | 0.15 (0.09 to 0.27) | <.001 |
| PSS-10 high (≥20) | 0.31 (0.19 to 0.52) | <.001 | 0.20 (0.12 to 0.36) | <.001 |
| QSC-R10 high (>14) | 0.39 (0.24 to 0.62) | <.001 | 0.24 (0.14 to 0.41) | <.001 |
| Obesity (BMI ≥30) | 0.88 (0.69 to 1.11) | .40 | 0.88 (0.64 to 1.21) | .60 |
| PSQI poor sleep (>5) | 0.45 (0.27 to 0.73) | <.001 | 0.33 (0.20 to 0.56) | <.001 |
| SBQ high (≥56 hours/week) | 0.59 (0.36 to 0.96) | .03 | 0.43 (0.26 to 0.71) | <.001 |

**Notes:** ORs estimated from adjusted generalized estimating equation models. Abbreviations: BMI, body mass index; CI, confidence interval; OR, odds ratio; PSQI, Pittsburgh Sleep Quality Index; PSS-10, Perceived Stress Scale; QSC-R10, Questionnaire on Stress in Cancer; SBQ, Sedentary Behaviour Questionnaire; WHO-5, World Health Organization 5-item Well-Being Index.

**Table S7. Responder analyses at 6 and 12 months using pre-specified thresholds**

| **Variable** | **Time point (months)** | **n** | **Responders (n)** | **Proportion** | **SE** | **95% CI** |
| --- | --- | --- | --- | --- | --- | --- |
| ΔWHO-5 ≥10 | 6 | 108 | 53 | 0.49 | 0.05 | 0.40 to 0.59 |
|  | 12 | 108 | 47 | 0.44 | 0.05 | 0.34 to 0.53 |
| WHO-5 low to high (>50) | 6 | 53 | 23 | 0.43 | 0.07 | 0.30 to 0.57 |
|  | 12 | 52 | 25 | 0.48 | 0.07 | 0.34 to 0.62 |
| ΔSWLS ≥5 | 6 | 110 | 23 | 0.21 | 0.04 | 0.13 to 0.29 |
|  | 12 | 108 | 31 | 0.29 | 0.04 | 0.20 to 0.37 |
| ΔPSS-10 ≤−4 | 6 | 107 | 39 | 0.36 | 0.05 | 0.27 to 0.46 |
|  | 12 | 106 | 46 | 0.43 | 0.05 | 0.34 to 0.53 |
| PSS-10 high to low (<20) | 6 | 38 | 12 | 0.32 | 0.08 | 0.17 to 0.46 |
|  | 12 | 40 | 20 | 0.50 | 0.08 | 0.35 to 0.65 |
| ΔQSC-R10 ≤−5 | 6 | 114 | 47 | 0.41 | 0.05 | 0.32 to 0.50 |
|  | 12 | 111 | 56 | 0.50 | 0.05 | 0.41 to 0.60 |
| QSC-R10 high to low (≤14) | 6 | 63 | 25 | 0.40 | 0.06 | 0.28 to 0.52 |
|  | 12 | 61 | 32 | 0.52 | 0.06 | 0.40 to 0.65 |
| ΔB-IPQ ≤−7 | 6 | 110 | 40 | 0.36 | 0.05 | 0.27 to 0.45 |
|  | 12 | 108 | 54 | 0.50 | 0.05 | 0.41 to 0.59 |
| ΔFACIT-F ≥3 | 6 | 108 | 55 | 0.51 | 0.05 | 0.41 to 0.60 |
|  | 12 | 109 | 62 | 0.57 | 0.05 | 0.48 to 0.66 |
| ΔSF-12 PCS ≥3 | 6 | 114 | 50 | 0.44 | 0.05 | 0.35 to 0.53 |
|  | 12 | 111 | 60 | 0.54 | 0.05 | 0.45 to 0.63 |
| ΔSF-12 MCS ≥3 | 6 | 114 | 48 | 0.42 | 0.05 | 0.33 to 0.51 |
|  | 12 | 111 | 53 | 0.48 | 0.05 | 0.38 to 0.57 |
| ΔWeight ≤−3% | 6 | 119 | 55 | 0.46 | 0.05 | 0.37 to 0.55 |
|  | 12 | 115 | 52 | 0.45 | 0.05 | 0.36 to 0.54 |
| PSQI high to low (≤5) | 6 | 85 | 22 | 0.26 | 0.05 | 0.17 to 0.35 |
|  | 12 | 80 | 28 | 0.35 | 0.05 | 0.25 to 0.45 |

**Notes:** Responder analyses based on categorical shifts to favourable categories (e.g., WHO-5 low to high) include only participants in the unfavourable category at baseline. CI = confidence interval; SE = standard error. Definitions and source references for thresholds are provided in the Supplementary Methods/Outcome Measure References section. Abbreviations: B-IPQ, Brief Illness Perception Questionnaire; CI, confidence interval; FACIT-F, Functional Assessment of Chronic Illness Therapy–Fatigue; PSQI, Pittsburgh Sleep Quality Index; PSS-10, Perceived Stress Scale; QSC-R10, Questionnaire on Stress in Cancer; SF-12 PCS/MCS, 12-item Short Form Health Survey Physical/Mental Component Summary; SWLS, Satisfaction With Life Scale; WHO-5, World Health Organization 5-item Well-Being Index.

**Table S8. Associations Between Changes in Candidate Factors and Changes in Outcomes From Baseline to 6 Months**

| **Change predictor (Δ baseline to 6)** | **ΔWHO-5**  **β (95% CI)** | ***P* value** | **ΔSWLS**  **β (95% CI)** | ***P* value** | **ΔPSS-10**  **β (95% CI)** | ***P* value** | **ΔQSC-R10**  **β (95% CI)** | ***P* value** |
| --- | --- | --- | --- | --- | --- | --- | --- | --- |
| Δ B-IPQ | −0.61  (−0.84 to −0.37) | <.001 | −0.20  (−0.29 to −0.10) | <.001 | 0.25  (0.18 to 0.33) | <.001 | 0.41  (0.31 to 0.51) | <.001 |
| Δ PSS-10 | −1.7  (−2.2 to −1.3) | <.001 | −0.49  (−0.69 to −0.28) | <.001 | — | — | — | — |
| Δ QSC-R10 | −0.80  (−1.1 to −0.46) | <.001 | −0.30  (−0.43 to −0.17) | <.001 | — | — | — | — |
| Δ FACIT-F | 0.93  (0.64 to 1.2) | <.001 | 0.30  (0.17 to 0.42) | <.001 | −0.39  (−0.48 to −0.31) | <.001 | −0.62  (−0.76 to −0.48) | <.001 |
| Δ PSQI | −2.2  (−3.1 to −1.3) | <.001 | −0.82  (−1.2 to −0.48) | <.001 | 0.75  (0.43 to 1.1) | <.001 | 1.4  (0.92 to 1.8) | <.001 |
| Δ SF-12 PCS | 0.42  (0.05 to 0.79) | .027 | 0.14  (0.01 to 0.28) | .038 | −0.18  (−0.30 to -0.05) | .006 | −0.53  (−0.68 to −0.38) | <.001 |
| Δ SF-12 MCS | 0.94  (0.64 to 1.20) | <.001 | 0.32  (0.20 to 0.43) | <.001 | −0.36  (−0.45 to −0.27) | <.001 | −0.44  (−0.60 to −0.29) | <.001 |
| Δ SBQ | −0.10  (−0.22 to 0.03) | .12 | −0.06  (−0.11 to −0.02) | .005 | 0.05  (0.01 to 0.10) | .014 | 0.13  (0.07 to 0.20) | <.001 |
| Δ MOS-SSS | 8.2  (3.8 to 13) | <.001 | 3.3  (1.6 to 5.0) | <.001 | −2.4  (−4.0 to −0.77) | .004 | −2.4  (−4.9 to −0.02) | 0.049 |
| Δ REAP-S | 1.1  (0.23to 1.9) | .013 | 0.40  (0.07 to 0.72) | .018 | −0.48  (−0.76 to −0.20) | <.001 | −0.77  (−1.2 to −0.35) | <.001 |
| Δ BMI | −0.65  (−1.6 to 0.29) | .2 | −0.20  (−0.57 to 0.16) | .3 | 0.13  (−0.19 to 0.45) | .4 | 0.57  (0.07 to 1.1) | .026 |
| Δ Weight | −0.11  (−0.27 to 0.04) | .14 | −0.04  (−0.09 to 0.02) | .2 | 0.02  (−0.03 to 0.07) | .5 | 0.09  (0.02 to 0.17) | .020 |
| Δ Drink score | −0.95  (−2.8 to 0.89) | .3 | −0.56  (−1.3 to 0.13) | .11 | −0.01  (−0.65 to 0.63) | >.9 | 0.70  (−0.23 to 1.6) | .14 |
| Δ Binge score | −3.5  (−6.6 to −0.33) | .030 | −0.63  (−1.9 to 0.59) | .3 | 0.70  (−0.40 to 1.8) | .2 | 0.14  (−1.4 to 1.7) | .9 |
| Δ FACIT–SP-12 | 1.1  (0.70 to 1.5) | <.001 | 0.48  (0.32 to 0.63) | <.001 | −0.36  (−0.50 to −0.22) | <.001 | −0.39  (−0.61 to −0.16) | .001 |
| Δ Cannabis score | 2.9  (−1.9 to 7.7) | .2 | 0.18  (−1.4 to 1.7) | .8 | −0.33  (−2.0 to 1.3) | .7 | 0.35  (−1.8 to 2.4) | .7 |

**Notes:** β coefficients represent associations between change in the candidate factor (follow-up minus baseline) and change in the outcome (follow-up minus baseline) from baseline to 6 months, estimated from adjusted models. Models adjusted for age, sex, relationship status, Charlson Comorbidity Index, psychotropic medication use, and baseline outcome values. “—” indicates not estimated because the change predictor is the same construct as the outcome change. Abbreviations: B-IPQ, Brief Illness Perception Questionnaire; BMI, body mass index; CI, confidence interval; FACIT-F, Functional Assessment of Chronic Illness Therapy–Fatigue; GEE, generalized estimating equations; MOS-SSS, Medical Outcomes Study Social Support Survey; PSQI, Pittsburgh Sleep Quality Index; PSS-10, Perceived Stress Scale; QSC-R10, Questionnaire on Stress in Cancer; REAP-S, Rapid Eating Assessment for Participants–Shortened; SBQ, Sedentary Behaviour Questionnaire; SF-12 PCS/MCS, 12-item Short Form Health Survey Physical/Mental Component Summary; SWLS, Satisfaction With Life Scale; WHO-5, World Health Organization 5-item Well-Being Index.

**Table S9. Associations Between Changes in Candidate Factors and Changes in Outcomes From Baseline to 12 Months**

| **Change predictor (Δ baseline to 12)** | **ΔWHO-5**  **β (95% CI)** | ***P* value** | **ΔSWLS**  **β (95% CI)** | ***P* value** | **ΔPSS-10**  **β (95% CI)** | ***P* value** | **ΔQSC-R10**  **β (95% CI)** | ***P* value** |
| --- | --- | --- | --- | --- | --- | --- | --- | --- |
| Δ B-IPQ | −0.50  (−0.75 to −0.26) | <.001 | −0.19  (−0.27 to −0.11) | <.001 | 0.19  (0.11 to 0.28) | <.001 | 0.42  (0.32 to 0.52) | <.001 |
| Δ PSS-10 | −1.6  (−2.1 to −1.1) | <.001 | −0.47  (−0.65 to −0.29) | <.001 | — | — | — | — |
| Δ QSC-R10 | −1.2  (−1.5 to −0.81) | <.001 | −0.37  (−0.48 to −0.25) | <.001 | — | — | — | — |
| Δ FACIT-F | 1.3  (1.0 to 1.6) | <.001 | 0.23  (0.11 to 0.35) | <.001 | −0.35  (−0.46 to −0.25) | <.001 | −0.60  (−0.73 to −0.46) | <.001 |
| Δ PSQI | −2.8  (−3.7 to −1.9) | <.001 | −0.72  (−1.0 to −0.42) | <.001 | 0.70  (0.39 to 1.0) | <.001 | 1.4  (0.99 to 1.8) | <.001 |
| Δ SF-12 PCS | 0.60  (0.28 to 0.92) | <.001 | 0.12  (0.02 to 0.23) | .025 | −0.12  (−0.23 to -0.01) | .035 | −0.39  (−0.53 to −0.24) | <.001 |
| Δ SF-12 MCS | 0.81  (0.47 to 1.10) | <.001 | 0.21  (0.10 to 0.33) | <.001 | −0.28  (−0.39 to −0.17) | <.001 | −0.30  (−0.47 to −0.14) | <.001 |
| Δ SBQ | −0.17  (−0.31 to −0.03) | .016 | −0.06  (−0.11 to −0.02) | .008 | 0.03  (−0.02 to 0.08) | .3 | 0.12  (0.05 to 0.18) | <.001 |
| Δ MOS-SSS | 8.5  (3.6 to 13) | <.001 | 2.7  (1.2 to 4.2) | <.001 | −1.9  (−3.7 to −0.13) | .035 | −4.3  (−6.6 to −2.0) | <.001 |
| Δ REAP-S | 1.6  (0.65 to 2.6) | .001 | 0.39  (0.06 to 0.72) | .022 | −0.42  (−0.75 to −0.08) | .015 | −0.75  (−1.2 to −0.30) | .001 |
| Δ BMI | −1.0  (−1.8 to −0.25) | .010 | −0.20  (−0.47 to 0.06) | .13 | 0.04  (−0.24 to 0.33) | .8 | 0.89  (0.57 to 1.2) | <.001 |
| Δ Weight | −0.17  (−0.30 to −0.04) | .010 | −0.03  (−0.07 to 0.01) | .2 | 0.00  (−0.04 to 0.05) | .9 | 0.14  (0.08 to 0.19) | <.001 |
| Δ Drink score | −3.1  (−5.6 to −0.57) | .017 | −0.88  (−1.7 to −0.05) | .037 | 1.2  (0.33 to 2.1) | .008 | 1.3  (0.15 to 2.4) | .027 |
| Δ Binge score | −3.3  (−5.5 to −0.98) | .005 | −0.50  (−1.3 to 0.28) | .2 | 0.97  (0.2 to 1.7) | .014 | 1.2  (0.08 to 2.3) | .035 |
| Δ FACIT–SP-12 | 1.3  (1.0 to 1.6) | <.001 | 0.36  (0.25 to 0.47) | <.001 | −0.31  (−0.43 to −0.19) | <.001 | −0.50  (−0.66 to −0.34) | <.001 |
| Δ Cannabis score | −6.5  (−11 to −1.5) | .011 | −1.7  (−3.3 to −0.12) | .035 | 2.8  (1.2 to 4.5) | .001 | 3.0  (0.66 to 5.3) | .012 |

**Notes:** β coefficients represent associations between change in the candidate factor (follow-up minus baseline) and change in the outcome (follow-up minus baseline) from baseline to 12 months, estimated from adjusted models. Models adjusted for age, sex, relationship status, Charlson Comorbidity Index, psychotropic medication use, and baseline outcome values. “—” indicates not estimated because the change predictor is the same construct as the outcome change. Abbreviations: B-IPQ, Brief Illness Perception Questionnaire; BMI, body mass index; CI, confidence interval; FACIT-F, Functional Assessment of Chronic Illness Therapy–Fatigue; FACIT-SP-12, Functional Assessment of Chronic Illness Therapy–Spiritual Well-Being Scale; GEE, generalized estimating equations; MOS-SSS, Medical Outcomes Study Social Support Survey; PSQI, Pittsburgh Sleep Quality Index; PSS-10, Perceived Stress Scale; QSC-R10, Questionnaire on Stress in Cancer; REAP-S, Rapid Eating Assessment for Participants–Shortened; SBQ, Sedentary Behaviour Questionnaire; SF-12 PCS/MCS, 12-item Short Form Health Survey Physical/Mental Component Summary; SWLS, Satisfaction With Life Scale; WHO-5, World Health Organization 5-item Well-Being Index.

**Table S10. Effect modification by baseline psychological characteristics: time × moderator interactions (exploratory)**

| **Baseline moderator** | **Interaction term** | **WHO-5 interaction β (95% CI)** | ***P* value** | **PSS-10 interaction β (95% CI)** | ***P* value** | **SWLS interaction β (95% CI)** | ***P* value** |
| --- | --- | --- | --- | --- | --- | --- | --- |
| Perceived stress (PSS-10) | 6 months × PSS-10 | 0.27  (−0.08 to 0.61) | .13 | −0.25  (−0.36 to −0.15) | <.001 | 0.20  (0.01 to 0.40) | .043 |
|  | 12 months × PSS-10 | 0.63  (0.17 to 1.10) | .007 | −0.43  (−0.57 to −0.29) | <.001 | 0.19  (0.00 to 0.38) | .049 |
| Illness perceptions (B-IPQ) | 6 months × B-IPQ | 0.11  (−0.13 to 0.36) | .4 | 0.01  (−0.08 to 0.10) | .8 | 0.08  (−0.01 to 0.18) | .086 |
|  | 12 months × B-IPQ | 0.13  (−0.10 to 0.37) | .3 | −0.06  (−0.15 to 0.04) | .2 | 0.11 (0.02 to 0.20) | .014 |
| Well-being (WHO-5) | 6 months × WHO-5 | −0.20  (−0.32 to −0.09) | <.001 | 0.01  (−0.03 to 0.05) | .6 | −0.03  (−0.08 to 0.02) | .3 |
|  | 12 months × WHO-5 | −0.28  (−0.43 to −0.13) | <.001 | 0.04  (−0.01 to 0.10) | .11 | −0.05  (−0.11 to 0.00) | .059 |
| Life satisfaction (SWLS) | 6 months × SWLS | 0.09  (−0.27 to 0.44) | .6 | 0.03  (−0.10 to 0.15) | .7 | −0.27  (−0.44 to −0.11) | .001 |
|  | 12 months × SWLS | −0.33  (−0.80 to 0.13) | .2 | 0.13  (−0.01 to 0.28) | .063 | −0.35  (−0.52 to −0.19) | <.001 |

**Notes:** β coefficients represent interaction terms between time (6 or 12 months) and baseline moderator values from adjusted generalized estimating equation models. All models adjusted for age, sex, comorbidity, relationship status, psychotropic medication use, and baseline outcome values. Abbreviations: B-IPQ, Brief Illness Perception Questionnaire; CI, confidence interval; PSS-10, Perceived Stress Scale; QSC-R10, Questionnaire on Stress in Cancer; SWLS, Satisfaction With Life Scale; WHO-5, World Health Organization 5-item Well-Being Index.
